# NICD3 mediates pro-angiogenic effects through SMAD3/TGFBI axis in colorectal cancer

**DOI:** 10.1038/s41419-026-08939-z

**Published:** 2026-06-15

**Authors:** Rui An, Ruihao Yu, Xinxin Wen, Qingyang Ma, Kelei Mao, Qi Zhang, Liyuan Mao, Xiaoyan Feng, Fang Xin, Ping Liu, Yijia Wang, Jiaqi Zhang, Haixia Huang, Xiaodong Yan, Wei Wang

**Affiliations:** 1 Department of Physiology and Pathophysiology, School of Basic Medical Sciences, Capital Medical University, Beijing, China; 2 School of Basic Medical Sciences, Capital Medical University, Beijing, China; 3 Laboratory for Clinical Medicine, Capital Medical University, Beijing, China; 4 School of Clinical Medicine, Jining Medical University, Jining, China

**Keywords:** Colon cancer, Tumour angiogenesis

## Abstract

The consensus molecular subtype 4 (CMS4) of colorectal cancer (CRC) represents an aggressive, mesenchymal phenotype associated with the poorest clinical prognosis. Although angiogenesis is fundamental to CRC progression, current anti-VEGF therapies are frequently compromised by primary or acquired resistance, creating an urgent need to identify alternative vascular drivers. NOTCH3 is significantly enriched in CMS4 CRC, yet its direct role in modulating the tumor vasculature remains elusive. Here, integrating multi-center transcriptomic datasets with clinical validation, we identified NOTCH3 as a critical engine of CRC angiogenesis. In Stage IV clinical samples, NOTCH3 expression correlated significantly with microvessel density (MVD). Functionally, overexpression of the NOTCH3 intracellular domain (NICD3) dramatically enhanced endothelial tube formation and migration in vitro, while driving robust tumor vascularization in xenograft models. Through transcriptomic profiling, we identified the secreted factor transforming growth factor-beta-induced protein (TGFBI) as the essential downstream effector. Mechanistically, we demonstrate that NICD3 physically interacts with and upregulates SMAD3, thereby facilitating the direct transcriptional activation of TGFBI. Crucially, to translate these mechanistic insights, we utilized molecular docking and drug screening to identify the flavonoid Hesperidin (HES) as a potent binder of TGFBI. HES treatment effectively abolished NICD3-driven angiogenesis and suppressed tumor progression in vivo. Collectively, our findings characterize a novel NICD3/SMAD3/TGFBI signaling axis as a key vulnerability in CRC and propose HES as a promising precision therapeutic strategy for CRC patients with features of CMS4.

## Introduction

Colorectal cancer (CRC) remains a leading cause of global cancer mortality, primarily driven by distant metastasis and therapeutic resistance [1–4]. The Consensus Molecular Subtypes (CMS) classification has fundamentally reshaped our understanding of CRC heterogeneity. Among these, the mesenchymal CMS4 subtype (approximately 23%) represents the most aggressive phenotype, characterized by extensive stromal infiltration, activation of the TGF-β pathway, and chemoresistance [5–7]. Crucially, CMS4 tumors exhibit a profound angiogenic signature distinct from other subtypes, suggesting that their vascular network is maintained by unique molecular drivers that foster a hostile, metastatic microenvironment [8].

While targeting angiogenesis with agents such as Bevacizumab (Bev) is standard-of-care, its efficacy is severely limited by intrinsic resistance and the activation of alternative angiogenic pathways [9, 10]. This is particularly evident in CMS4 tumors, where the dense stromal compartment limits drug penetration and provides non-VEGF pro-angiogenic signals [11, 12]. Therefore, targeting VEGF alone is insufficient. Unraveling the stroma-derived or mesenchymal-associated angiogenic mechanisms is imperative to overcome resistance and improve outcomes for this high-risk patient population.

The Notch signaling pathway serves as a master regulator of cell-fate determination, yet its four paralogues exhibit highly divergent roles in tumorigenesis [13–15]. In CRC, NOTCH3 displays a strikingly distinct pathobiological function compared with NOTCH1: it is selectively amplified and overexpressed in the mesenchymal CMS4 subtype and is tightly linked to the maintenance of cancer stemness and distant metastasis [16]. Furthermore, studies utilizing mouse models that recapitulate human CMS4 features have identified significant Notch3 pathway activation [17], thereby establishing NOTCH3 as a compelling molecular driver and potential therapeutic target in CMS4 CRC. Classically, Notch3 signaling is transduced through the canonical pathway, where the cleaved NOTCH3 intracellular domain (NICD3) translocates to the nucleus and forms a transcriptional complex with RBPJ (CSL) to activate downstream target genes [18]. However, therapeutic strategies strictly targeting this canonical axis have shown limited clinical efficacy, suggesting the involvement of alternative mechanisms [13, 14, 19]. Emerging evidence has revealed that NOTCH3 can operate independently of the canonical RBPJ/CSL-dependent transcriptional program by engaging in non-canonical crosstalk with core signaling cascades, including TGF-β/SMAD and Wnt/β-catenin pathways. This non-canonical interplay profoundly reprograms the tumor secretome, driving the secretion of pro-angiogenic and stroma-remodeling factors such as transforming growth factor-beta-induced protein (TGFBI), POSTN, and IL11, thereby sustaining an aggressive phenotype and fostering therapeutic resistance [20–22]. However, whether NOTCH3 functions as an upstream orchestrator of the angiogenic switch in CRC independently of the classical VEGF axis remains a critical unresolved question—a knowledge gap that the present study specifically aims to address.

In this study, we identify a novel NICD3/SMAD3/TGFBI axis as a potent driver of CRC angiogenesis. We demonstrate that NICD3 transcriptionally upregulates TGFBI via SMAD3 recruitment, thereby remodeling the vascular microenvironment. Furthermore, utilizing structure-based virtual screening, we repurposed the natural flavonoid Hesperidin (HES) as a specific binder of TGFBI that inhibits its pathological function. Our findings provide the first mechanistic evidence linking NOTCH3 to neovascularization and propose a targeted strategy to dismantle the angiogenic network in CRC with features of CMS4.

## Materials and methods

### Cell culture and treatment

The CRC cell lines (HCT116, SW620, MC38) and the human embryonic kidney 293T (HEK293T) cell line were purchased from Procell Life Science & Technology Company (Wuhan, China). Human umbilical vein endothelial cells (HUVECs) were obtained from the Cell Resource Center, Peking Union Medical College (1101HUM-PUMC000437). HCT116 cells were cultured in McCoy’s 5A medium (Gibco, USA) and the other cell lines were cultured in high-glucose Dulbecco’s modified Eagle’s medium (Gibco), supplemented with 10% FBS (Gibco) at 37 °C in a humidified atmosphere containing 5% CO_2_.

For rescue experiments, recombinant human TGFBI (rTGFBI, MedChemExpress, HY-P74525A) was reconstituted according to the manufacturer’s instructions. HUVECs were treated with rTGFBI at a final concentration of 50 μg/mL for 24 h before being subjected to tube formation or migration assays [23]. An equivalent volume of the protein solvent was used as the vehicle control.

### Transfection and stable cell line generation

The coding sequence of human NICD3 was cloned into the pHBLV-CMV-MCS-EF1-ZsGreen-T2A-puro vector by multiple cloning sites, which was provided by Han Heng Biology (Shanghai, China). HCT116 and SW620 cells were infected with the lentivirus, and stable cells were generated by puromycin selection at 48 h post-transfection.

SiRNAs were purchased from Han Heng Biology (Shanghai, China). The sequences of the siRNAs were as follows:

si-NOTCH3 #1, 5′-GAUGACACAUCAGCUAGCAUCAUCU-3′;

si-TGFBI #2, 5′-GAGACAAUCGCUUUAGCAUGC-3′;

si-TGFBI#3, 5′-GCUUGAGAUCUUCAAACAATT-3′;

si-SMAD3 #1, 5′-GGCCAACAUAGGCAAAUGATT-3′;

si-SMAD3 #3, 5′-GCCACCUUCAAUUGGUACUTT-3′;

negative control (NC), 5′-UUCUCCGAACGUGUCACGUTT-3′.

For knocking down NOTCH3, TGFBI, or SMAD3 expression, CRC cells were separately transfected with 50 nM of each siRNA using Lipo 3000 transfection reagent (Thermo Fisher Scientific, USA).

### Subcutaneous xenograft model

The male BALB/c nude mice were obtained from Beijing Vital River Laboratory Animal Technology Co., Ltd (Beijing, China). To investigate angiogenesis, 4 × 10^6^ SW620 cells or 6 × 10^6^ HCT116 cells (NC or NICD3) with 0.1 mL PBS were subcutaneously transplanted into the right flanks of 4-week-old nude mice.

When the tumors reached a palpable size (approximately 100 mm^3^), the mice were randomly assigned into five treatment cohorts: (1) NC + Vehicle, (2) NC + HES, (3) NICD3 + Vehicle, (4) NICD3 + HES, and (5) NICD3 + Bev. For the therapeutic groups, Hesperidin (HES, 60 mg/kg) (MedChemExpress, HY-15337) was administered daily via intraperitoneal (i.p.) injection. As a positive control, Bevacizumab (Bev, 5 mg/kg) (MedChemExpress, HY-P9906A) was administered twice weekly via i.p. injection. Control groups received an equivalent volume of vehicle.

Tumor volumes were calculated at the indicated time intervals, using the formula: volume = 0.5 × length × width^2^. The mice were euthanized, and subcutaneous tumors were harvested for further analysis after 16 days. All animal experiments were conducted under the approval of the Ethics Committee of the Capital Medical University (AEEI-2025-611). The maximal tumor burden (volume ≤1500 mm³) specified in the approved protocol was not exceeded at any point during the study.

### Multiplex immunofluorescence staining and analysis

Multiplex immunofluorescence staining was performed on a formalin-fixed, paraffin-embedded human CRC tissue microarray (Shanghai Outdo Biotech, TMA: HCo1-Ade090CS4-01, China) using a commercial kit (Recordbio Biological Technology, RC0086-67RM, China). Following deparaffinization and rehydration, antigen retrieval was conducted using EDTA buffer (pH 9.0) with microwave heating. Sections were then incubated with 3% hydrogen peroxide to quench endogenous peroxidase activity and blocked with 3% bovine serum albumin. Primary antibodies against NOTCH3 (Proteintech, 55114-1-AP) and CD31(Abclonal, A19014) were applied and incubated overnight at 4 °C. Subsequently, signals were amplified using appropriate secondary antibodies coupled with tyramide signal amplification (TSA) and Opal Fluorophores. Nuclei were counterstained with DAPI before mounting.

Whole-slide imaging was carried out using a slide viewer system. Quantitative analysis of microvessel density (MVD) was performed with the VesselJ plugin in ImageJ software.

### Public database analysis

Data on CMS and NOTCH3 mRNA expression in CRC tissues and associated clinical information were retrieved from The Cancer Genome Atlas (TCGA) and Gene Expression Omnibus (GEO) databases. Patients were stratified into high- and low-expression groups based on NOTCH3 mRNA levels using the upper and lower quartiles as the cutoff. Differential expression analysis was subsequently performed between these groups to identify differentially expressed genes. Gene Ontology enrichment analysis (http://www.geneontology.org) on identifying differentially expressed genes was performed. (*P*-value < 0.05 as the cutoff value).

The correlation between NOTCH3 and CD31 in CRC was analyzed using the cBioPortal database (https://www.cbioportal.org/). Overall and relapse-free survival curves of TCGA patients were generated using the Kaplan-Meier Plotter, and statistical significance was assessed by the log-rank test.

### Western blot analysis

Cells were rinsed with Phosphate-buffered saline (PBS) and lysed using RIPA buffer (Applygen, China) at 4 °C for 40 min. Equal amounts of protein lysates were separated by SDS-PAGE and transferred to PVDF membranes (Merck Millipore, Germany). Subsequently, membranes were blocked with 10% non-fat milk powder and then incubated with primary antibodies against NOTCH3 (CST, 5276S), TGFBI (Abcam, ab170874), Phospho-SMAD3 (Ser423/425) (CST, 9520S), SMAD3 (Abcam, ab208182), Phospho-SMAD2 (S465/S467) (ABclonal, AP1342), SMAD2 (ABclonal, A19114), SMAD4 (ABclonal, A21487), Flag-tag (CST, 14793S), Myc-tag (CST, 2276S), β-actin (ABclonal, AC004). For protein detection, ECL reagents A and B (Thermo Fisher Scientific) were mixed at a 1:1 (v/v) ratio and applied to the membrane surface, followed by 1-min incubation at room temperature. Chemiluminescence signals were captured using an Amersham ImageQuant 800 imaging system (Cytiva, USA). Digital images were acquired using automatic exposure settings to ensure a linear detection range. Target protein bands were quantified based on their grayscale values using ImageJ software and normalized to β-actin as an internal control.

### Co-immunoprecipitation (Co-IP) assay

Cells were harvested and washed with ice-cold PBS, then lysed in RIPA buffer (50 mM HEPES, pH 7.4, 150 mM NaCl, 1% NP-40, 1 mM EDTA) supplemented with 1× protease inhibitor cocktail (Roche, 04693116001, Switzerland). Cell debris was removed with centrifugation, and 10% of the supernatant was saved as input. Cell lysates were pre-cleared with Dynabeads™ Protein G (Thermo Fisher Scientific) for 20 min at 4 °C. Anti-NOTCH3 antibody (CST, 5276S) or Normal Rabbit IgG (CST, 2729S) was diluted in 200 μL PBST with Dynabeads™ Protein G, and incubated for 20 min at room temperature with flip. Samples were incubated with the mixture of antibodies and magnetic beads overnight at 4 °C. The immunoprecipitates were washed with RIPA buffer, recovered with SDS sample buffer and separated on SDS-PAGE, followed by western blotting using antibodies against SMAD3 (CST, 9523S), SMAD2 (ABclonal, A19114), and SMAD4 (ABclonal, A21487).

### RNA extraction and quantitative real-time PCR (RT-qPCR)

Total RNA was isolated from HCT116 and SW620 cells by FastPure Cell/Tissue Total RNA Isolation Kit V2 (Vazyme, China), following the manufacturer’s instructions, and its concentration and purity were determined using a NanoDrop One spectrophotometer (Thermo Fisher Scientific, USA). Reverse transcription utilized HiScript III RT SuperMix for qPCR (Vazyme). RT-qPCR was conducted in triplicate utilizing SYBR Green (Vazyme). Primers are listed in Table S1.

### Cleavage under targets and release using nuclease (CUT&RUN)-qPCR analysis

CUT&RUN assays were performed using the CUT&RUN-qPCR Kit (Vazyme, HD103) according to the manufacturer’s instructions. Briefly, 5 × 10^5^ cells were harvested and bound to Con A-coated beads. The cell-bead complexes were incubated overnight at 4 °C with a primary antibody against SMAD3 (Abcam, ab208182) or Normal Rabbit IgG (CST, 2729S) in prechilled antibody buffer. After incubation, samples were mixed with pG-MNase enzyme for 1 h. Following centrifugation, DNA was fragmented by adding CaCl_2_ for 2 h at room temperature, and the reaction was terminated by the addition of stop buffer. The purified DNA was then extracted and used as a template for qPCR analysis.

Three sets of primers (P1, P2, and P3) were designed to span the *TGFBI* promoter region to map the binding site. The recruitment levels of SMAD3 were quantified as the fold enrichment relative to the IgG control using the 2^−∆∆Ct^ method, ensuring that non-specific background noise was eliminated. Statistical significance was determined by an unpaired Student’s *t-*test based on three independent biological replicates. The specific primer sequences for CUT&RUN-qPCR are provided in Table S1.

### Dual luciferase reporter assay

The human *TGFBI* promoter region was cloned into the pmirGLO-basic vector. For the assay, HCT116-NC and HCT116-NICD3 cells were seeded and co-transfected with the pmirGLO-TGFBI reporter plasmid. At 48 h post-transfection, Firefly and Renilla luciferase activities were measured using the Dual-Luciferase Reporter Assay System (Promega, USA). The Firefly luciferase signal was normalized to the Renilla signal for each sample to account for transfection efficiency.

### HUVEC transwell migration

HUVEC migration was performed with a transwell system (Corning, USA) containing polycarbonate filters (diameter, 6.5 mm) with a pore size of 8 μm. The conditioned medium (CM) from HCT116 or SW620 cells was added to the lower chambers for 500 μL each. Harvested and resuspended HUVECs in 200 μL of serum-free DMEM were then seeded into the upper chambers (4 × 10^4^ cells/well). After incubation for 18-20 h, the cells were fixed with 4% paraformaldehyde and stained with 0.1% crystal violet solution. The cells were photographed, and the number of migrated HUVECs was counted.

### HUVEC tube formation

The CM for the endothelial tube formation assay was harvested from HCT116 or SW620 cells after serum-free medium treatment for 24 h. Briefly, HUVECs (2 × 10^5^ cells/well) suspended in 100 μL of CM were seeded in 96-well plates pre-coated with 30 μL of Matrigel. After 5 h of incubation, capillary-like tubes formed by HUVECs were observed and photographed, and the total branching length was counted and analyzed by ImageJ.

### Cell proliferation and cytotoxicity assay

Cell proliferation and cytotoxicity were assessed using CCK-8 kits (GlpBio, USA). The absorbance of the solution was measured on a microplate reader at 450 nm.

To measure cell proliferation, 100 μL HCT116 or SW620 cells were seeded in 96-well plates (4 × 10^3^ cells/well), allowing the cells to attach. After 24 h, 48 h, and 72 h, the cells were incubated with 10 μL CCK-8 solution at 37 °C for an additional 2 h before absorbance measurement.

For the cytotoxicity assay, 100 μL HCT116 or SW620 cells were seeded in 96-well plates (5 × 10^3^ cells/well). After 24 h, the cells were treated with various concentrations of drugs for 24 or 48 h, followed by the addition of 10 μL CCK-8 solution and incubation at 37 °C for 3 h before absorbance measurement.

### Enzyme-linked immunosorbent assay (ELISA)

The concentration of TGFBI in CRC cell-CM was detected by the human TGFBI ELISA Kit (Jingmei Biotechnology, JM-6789H1, China) according to the manufacturer’s instructions. In brief, 50 µL of standard solution or 1:5-diluted sample was added to wells pre-coated with capture antibody. One hundred microliters of enzyme-conjugated reagent was added to each well, followed by incubation at 37 °C for 1 h. After five washing cycles, 50 µL of Chromogenic Solution A and 50 µL of Chromogenic Solution B were dispensed into each well. Following gentle shaking to ensure thorough mixing, the wells were incubated in the dark at 37 °C for 15 min. Then, 50 µL of stop solution was added to each well. Finally, the absorbance was measured at 450 nm using an enzyme labeling instrument.

### 5D Label-free quantitative proteomics

To unbiasedly profile the NICD3-regulated secretome, CM from HCT116 cells were subjected to 5D label-free mass spectrometry (MS), performed by Shanghai Genechem Co., Ltd (Shanghai, China). Briefly, proteins extracted with SDT buffer were digested using the Filter-Aided Sample Preparation (FASP) method.

The resulting peptides were analyzed on a nanoElute LC system coupled to a timsTOF Pro mass spectrometer (Bruker) operating in PASEF mode, which incorporates the ion mobility dimension to enhance identification depth. MS data were processed using PaSER 2023 software against the UniProt human database. Both peptide and protein false discovery rates (FDR) were maintained at 1%. Differentially expressed proteins were identified using the criteria of fold change > 1.5 and *P* < 0.05. The comprehensive results of the MS analysis are provided in Table S2.

### Cellular thermal shift assay (CETSA)

The NICD3-overexpressing CRC cells were treated with or without HES (200 μM) for 24 h and then collected. Cell suspensions in PBS with protease inhibitor were divided into six equal parts and heated for 3 min at the indicated temperatures (37, 40, 43, 46, 49, and 52 °C). Afterward, cell lysis was achieved through two alternating cycles of 3 min in liquid nitrogen followed by 5 min at 25 °C. For quantification of TGFBI protein, the supernatant was collected by centrifugation at 15,000 rpm for 20 min and analyzed by western blotting.

### Drug affinity responsive target stability (DARTS)

The DARTS assay was performed to assess the physical interaction between HES and TGFBI [24, 25]. NICD3-overexpressing CRC cells were lysed in ice-cold non-denaturing lysis buffer. Lysates were cleared by centrifugation at 12,000 rpm for 10 min, and protein concentrations were determined using the BCA assay. Equal amounts of protein were incubated with HES (50, 100, or 200 μM) or DMSO (vehicle) for 30 min at room temperature with gentle rotation. Samples were then digested with Pronase (Beyotime, S3206) at a protease-to-protein ratio of 1:1000 for 30 min at room temperature. Proteolysis was halted by the addition of a protease inhibitor cocktail, followed by boiling in 5× SDS loading buffer. TGFBI stability was assessed by western blotting.

### Immunofluorescence

For the immunofluorescence assay, tumor tissues from a mouse xenograft model were fixed in 10% Formalin at room temperature, embedded in paraffin and sectioned into 20-μm-thick slices. The tissue sections were dewaxed and subjected to antigen retrieval, followed by blocking with 5% bovine serum albumin (Macklin, China) for 1 h and permeabilizing with 0.1% Triton X-100 in PBS at room temperature. Next, samples in a dark, humid chamber were incubated successively with anti-CD31 (Abcam, ab222783) at 4 °C overnight and fluorescent-labeled anti-rat IgG (Thermo Fisher Scientific, A-11006) at room temperature for 1 h. After being stained with DAPI (Beyotime, China) to visualize nuclei, the tissue sections were observed and captured using fluorescent microscopy (Thermo Fisher Scientific).

### RNA sequencing

Total RNA was extracted from stable cell lines overexpressing NICD3 or a control vector. Transcriptome sequencing was performed on the Illumina Novaseq platform in Shanghai Genechem Co., Ltd (Shanghai, China). Gene expression was quantified, and differential expression analysis was performed using DESeq2 (|log2(FoldChange)| ≥1, *p*_adj_ ≤ 0.05). Kyoto Encyclopedia of Genes and Genomes enrichment analysis of differentially expressed genes was conducted and visualized using R software.

### Molecular docking

Using the Protein Preparation Wizard module, hydrogen atoms were added, water molecules and ACT molecules were removed. The prepared protein structure was then subjected to binding site prediction using the SiteMap module. The 2D structures of compounds from the HY-L001P MCE Bioactive Compound Library were processed with the LigPrep module to add hydrogens and perform energy minimization, generating optimized 3D structures for subsequent virtual screening. Virtual screening was carried out using the Virtual Screening Workflow in Schrödinger. Compounds were initially screened in high-throughput virtual screening mode with the Glide module, and the top 15% of scored ligands were advanced to standard precision docking. From these, the top 15% were further evaluated using extra precision docking, resulting in a final selection of the top 200 ranked compounds.

### Statistical analysis

All statistical analyses and graphs were generated using GraphPad Prism, version 10.4 (GraphPad Software, USA). Data are presented as mean ± SD or mean ± SEM, as indicated in the figure legends. For comparisons between two groups, an unpaired Student’s *t* test was used for independent observations, while a paired Student’s *t-*test was employed for matched samples. Multiple group comparisons were analyzed using one-way or two-way ANOVA, followed by Sidak’s post-hoc test for specific inter-group differences. Normal distribution and homogeneity of variance were confirmed before parametric testing. Statistical significance is indicated as *P < 0.05, **P < 0.01, and ***P < 0.001.

## Results

### NOTCH3 upregulation, combined with angiogenesis, is associated with the prognosis of CRC patients

**Fig. 1:**
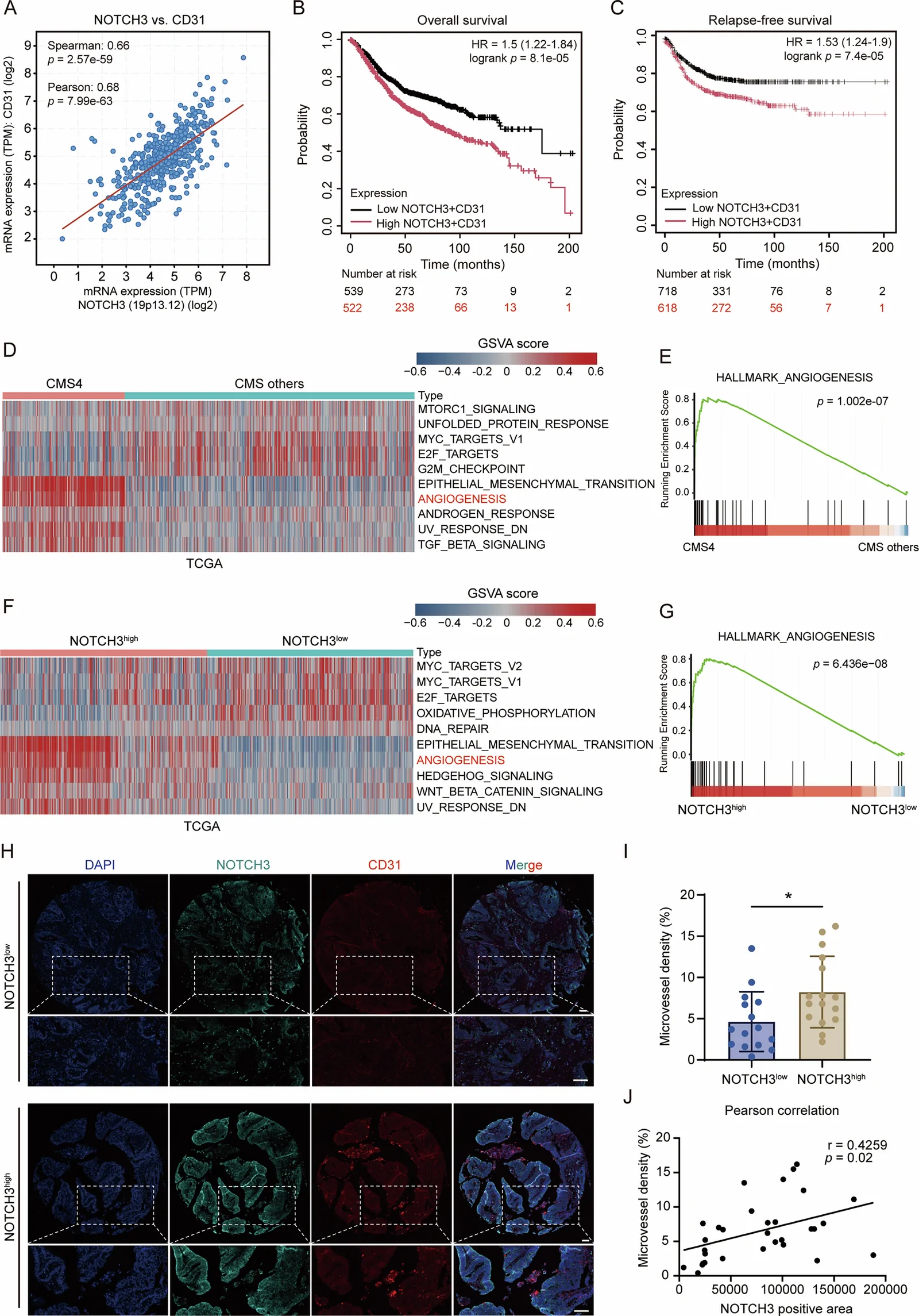
High NOTCH3 expression promotes angiogenesis and predicts poor prognosis in CRC. **A** Correlation analysis between NOTCH3 and CD31 mRNA expression based on the cBioPortal. (Spearman *r* = 0.66, *P* = 2.57e-59; Pearson *r* = 0.68, *P* = 7.99e-63). **B** The overall survival analysis and **C** relapse-free survival analysis of CRC patients based on the data from the TCGA database using the Kaplan-Meier Plotter. **D** GSVA and **E** GSEA enrichment analysis of CMS4 and CMS others in CRC patients from the TCGA dataset. **F** GSVA enrichment analysis and **G** GSEA enrichment analysis of NOTCH3^high^ and NOTCH3^low^ groups in CRC patients from the TCGA dataset. **H** Representative multiplexed immunofluorescence images of NOTCH3^high^ and NOTCH3^low^ tissues. Scale bar, 100 μm. **I** Histogram of MVD in NOTCH3^high^ and NOTCH3^low^ samples (Student’s *t-*test, *n* = 15–16). **J** Correlation between NOTCH3-positive area and MVD (Pearson *r* = 0.4259, *P* = 0.02). Data are presented as mean ± SD. Statistical significance was determined by the log-rank test (**B**, **C**) and Student’s *t-*test (I). *P < 0.05.

To investigate the relationship between NOTCH3 expression and angiogenesis in clinical CRC samples, we interrogated the cBioPortal database and found that NOTCH3 exhibited a significant positive correlation with the vascular endothelial marker CD31 (Fig. 1A). Furthermore, analysis of the Kaplan–Meier Plotter database revealed that patients with high co-expression of NOTCH3 and CD31 exhibited poorer overall survival and relapse-free survival (Fig. 1B, C). We next integrated transcriptomic data from CRC patients available in the TCGA and GEO databases. After classifying patients according to CMS, we observed that those with CMS4 exhibited significant enrichment of angiogenesis-related gene sets, as determined by Gene Set Variation Analysis (GSVA) and Gene Set Enrichment Analysis (GSEA) (Figs. 1D, E, and S1A, B). Subsequently, samples were stratified into NOTCH3^high^ and NOTCH3^low^ groups based on NOTCH3 expression levels. We also observed that NOTCH3^high^ CRC patients exhibited significant enrichment of angiogenesis-related gene sets, as determined by GSVA and GSEA (Figs. 1F, G and S1C, D).

To further validate the relationship between NOTCH3 and tumor angiogenesis, we performed multiplex immunofluorescence analysis on a tissue microarray comprising 31 stage IV CRC samples. CD31-labeled microvessel density (MVD) was used to assess the extent of angiogenesis [26, 27]. We identified a significant increase in MVD in NOTCH3^high^ tissues compared to NOTCH3^low^ tissues (Fig. 1H, I), along with a strong positive correlation between NOTCH3 expression and MVD (Fig. 1J). Collectively, these findings suggest that enhanced angiogenesis is a prominent feature of CRC patients with high NOTCH3 expression, which is associated with inferior clinical outcomes.

### NICD3 overexpression promotes CRC angiogenesis in vitro and in vivo

**Fig. 2:**
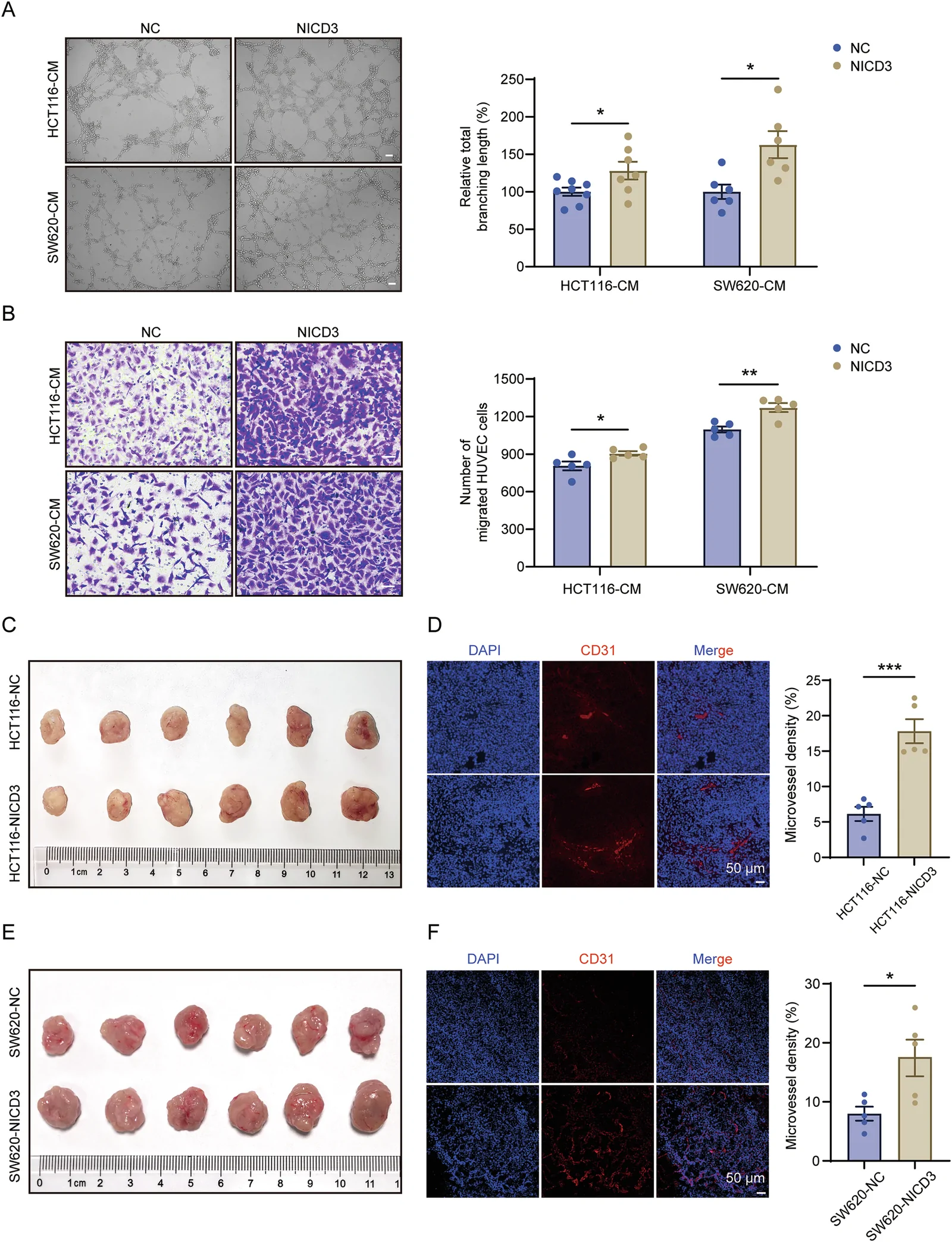
NICD3-driven angiogenesis in CRC. **A** Representative images of HUVEC tube formation with CM from the control and NICD3-overexpressing CRC cells (left panel). Quantitative analysis of the total branching length (*n* = 6–8, right panel). Scale bar, 100 μm. **B** Representative images of HUVEC migration with CM from the control and NICD3-overexpressing CRC cells (left panel). Quantitative analysis of migrated cells (*n* = 5, right panel). Scale bar, 50 μm. **C** Representative images of subcutaneous xenografts from nude mice injected with control and NICD3-overexpressing HCT116 cells (*n* = 6). **D** Representative immunofluorescence images (left panel) and quantification of CD31 expression in HCT116 xenografts (*n* = 5, right panel). Scale bar, 50 μm. **E** Representative images of subcutaneous xenografts from nude mice injected with control and NICD3-overexpressing SW620 cells (*n* = 6). **F** Representative immunofluorescence images (left panel) and quantification of CD31 expression in SW620 xenografts (*n* = 5, right panel). Scale bar, 50 μm. Data are presented as mean ± SD. Statistical significance was determined by Student’s *t-*test (**A**, **B**, **D**, **F**). *P < 0.05, **P < 0.01, ***P < 0.001.

To experimentally validate the above clinical findings, we performed cell-based and animal studies to investigate the functional role of NOTCH3 in tumor angiogenesis. Ectopic expression of NICD3, a key driver of Notch signaling, was established in HCT116 and SW620 CRC cells. Functional experiments demonstrated that conditioned medium (CM) from NICD3-overexpressing CRC cells significantly enhanced both tube formation and the migration capacity of HUVECs (Fig. 2A, B). Consistent with these pro-angiogenic effects in vitro, immunofluorescence analysis revealed a significant increase in MVD in xenografts derived from HCT116-NICD3 and SW620-NICD3 cells compared to controls (Fig. 2C–F). Notably, this enhanced angiogenesis occurred in the absence of a corresponding increase in tumor volume (Fig. S2A, B), prompting us to examine cell-autonomous effects. We found that NICD3 overexpression unexpectedly attenuated the proliferative capacity of CRC cells in vitro (Fig. S2C, D), suggesting that NOTCH3-driven tumor growth in vivo is primarily dependent on angiogenesis. The pro-angiogenic role of NICD3 was further validated in a syngeneic mouse model, where MC38-Nicd3 tumors similarly exhibited a significant increase in MVD (Fig. S2E). Collectively, these data demonstrate that NICD3 functions as a critical promoter of tumor angiogenesis in CRC.

### NICD3 drives tumor angiogenesis in CRC through transcriptional upregulation of TGFBI

**Fig. 3:**
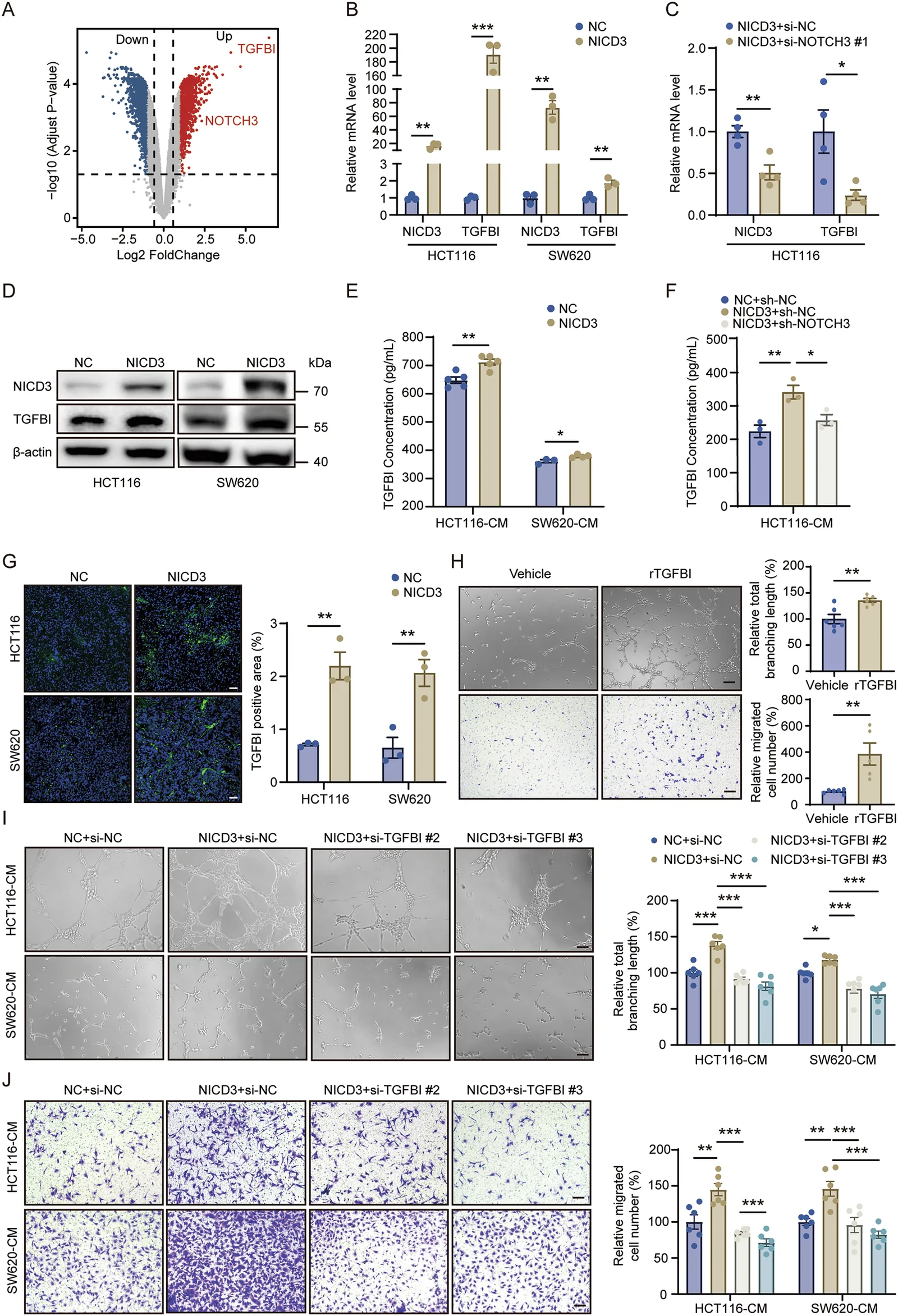
NICD3-overexpressing CRC cells promote tumor angiogenesis by upregulating TGFBI. **A** Volcano plot showing differentially expressed genes between control and NICD3-overexpressing HCT116 cells using RNA sequencing (*n* = 3). **B** The relative expression of TGFBI mRNA was detected in control and NICD3-overexpressing CRC cells (*n* = 3). **C** The relative expression of TGFBI mRNA was detected in control and NOTCH3-silencing CRC cells (*n* = 4). **D** Western blot analysis was performed to measure the levels of TGFBI in control and NICD3-overexpressing CRC cells. **E** TGFBI protein concentration in CM from control and NICD3-overexpressing cells was measured by ELISA (*n* = 3–5). **F** TGFBI protein concentration in CM from control, NICD3-overexpressing, and NICD3-overexpressing followed by NOTCH3 silencing HCT116 cells was measured by ELISA (*n* = 3). **G** Representative immunofluorescence images (left panel) and quantification of TGFBI expression in xenografts from control and NICD3-overexpressing CRC cells (*n* = 3, right panel). Scale bar, 50 μm. **H** Representative images of HUVEC tube formation and migration with CM from the control and rTGFBI-treated (50 μg/mL) HUVEC (left panel). Quantitative analysis of the total branching length and migrated cells (*n* = 5–6, right panel). Scale bar, 50 μm. **I** Representative images of HUVEC tube formation with CM from the control and TGFBI silencing CRC cells (left panel). Quantitative analysis of the total branching length (*n* = 6, right panel). Scale bar, 50 μm. **J** Representative images of HUVEC migration with CM from the control and TGFBI silencing CRC cells (left panel). Quantitative analysis of migrated cells (*n* = 6, right panel). Scale bar, 50 μm. Data are presented as mean ± SD. Statistical significance was determined by Student’s *t-*test (**B**, **C**, **E**, **G**, **H**) and one-way ANOVA (**F**, **I**, **J**). *P < 0.05, **P < 0.01, ***P < 0.001.

We next sought to investigate the underlying molecular mechanism by which NICD3 exerts this pro-angiogenic effect in CRC. We first conducted RNA sequencing on HCT116 cells stably overexpressing NICD3 relative to control cells. Transcriptomic analysis identified TGFBI as the most significantly upregulated gene (*p*_adj_ < 0.0001, |log2(FoldChange)| ≥ 5) (Fig. 3A). TGFBI is a well-defined downstream effector of TGF-β signaling, encoding a 68 kDa secreted protein that is deposited into the extracellular matrix. It is transcriptionally induced via both the canonical phospho-SMAD2/3 and non-canonical p38 MAPK pathways. Once secreted into the tumor microenvironment, TGFBI promotes malignant progression by facilitating tumor cell proliferation, invasion, and migration [28]. Notably, TGFBI promotes metastatic extravasation by activating αVβ5 integrin signaling, thereby disrupting the VE-cadherin-dependent endothelial barrier [29]. In the TCGA and GEO CRC cohorts, CMS4 patients displayed pronounced TGFBI upregulation relative to other subtypes. A significant positive correlation was also observed between NOTCH3 and TGFBI expression levels (Fig. S2F, G).

Given the established pro-angiogenic and pro-metastatic functions of TGFBI, we hypothesized that it may serve as a critical mediator of NICD3-driven angiogenesis. To validate this regulatory axis, we performed gain- and loss-of-function experiments. RT-qPCR analysis revealed that TGFBI mRNA levels were significantly upregulated following NICD3 overexpression (Fig. 3B), whereas NOTCH3 silencing effectively suppressed TGFBI induction (Fig. 3C), demonstrating that NICD3 modulates TGFBI at the transcriptional level. Consistently, western blot analysis confirmed a significant increase in TGFBI protein expression upon NICD3 overexpression in both HCT116 and SW620 cells (Fig. 3D). Given that TGFBI is a secreted protein, we examined its extracellular levels by ELISA and observed a significant increase in TGFBI levels in CM from NICD3-overexpressing cells (Fig. 3E). Importantly, NOTCH3 silencing reversed this elevation of TGFBI secretion (Fig. 3F). To unbiasedly profile the NICD3-regulated secretome, 5D label-free mass spectrometry (MS) analysis of the CM which revealed a marked elevation of secreted TGFBI upon NICD3 overexpression (Fig. S2H). Moreover, immunofluorescence staining of xenograft revealed substantial TGFBI accumulation in the stroma of NICD3-overexpressing tumors (Fig. 3G). Collectively, these results demonstrate that NICD3 drives both the secretion of TGFBI in vitro and its subsequent deposition in the tumor stroma in vivo.

We next investigated whether TGFBI is sufficient to independently drive angiogenesis. To this end, HUVECs were treated with recombinant human TGFBI (rTGFBI, 50 μg/mL) [23]. Functional assays demonstrated that exogenous rTGFBI significantly enhanced HUVEC tube formation and migration (Fig. 3H), identifying TGFBI as a potent and direct pro-angiogenic effector. Finally, we knocked down TGFBI expression in NICD3-overexpressing CRC cells (Fig. S2I) and found that TGFBI silencing markedly attenuated the enhanced tube-forming and migratory capacities of HUVECs induced by NICD3-overexpressing CRC cells-derived CM (Fig. 3I, J).

Collectively, these findings establish TGFBI as a key transcriptional target of NICD3 and a pivotal downstream effector mediating NICD3-induced angiogenesis in CRC.

### A non-canonical NICD3/SMAD3/TGFBI axis promotes tumor angiogenesis in CRC

**Fig. 4:**
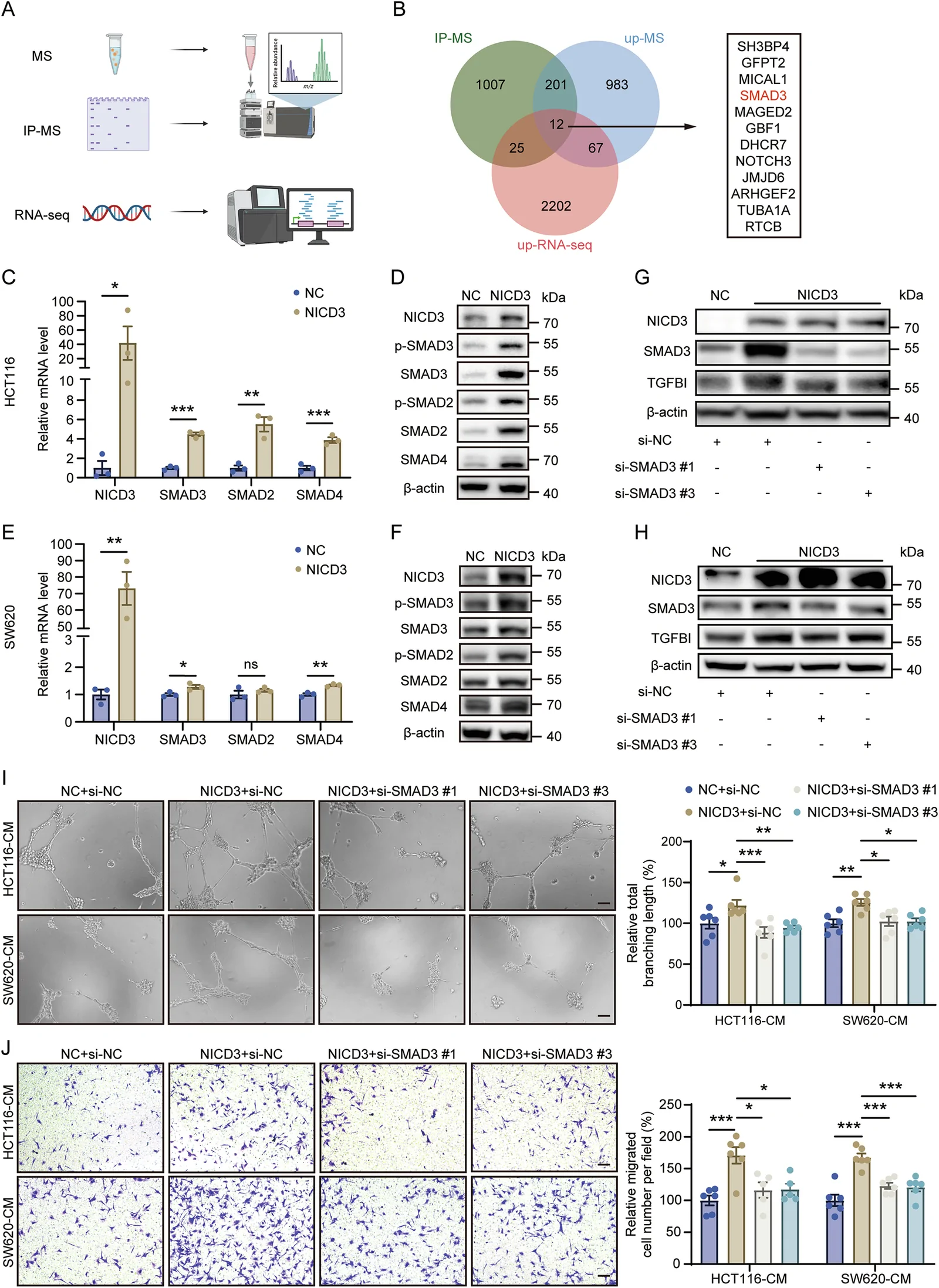
SMAD3 is identified as a critical mediator of NICD3-induced TGFBI expression to promote angiogenesis. **A** Schematic diagram of the screening for downstream molecules of NICD3. **B** Intersection analysis of up-regulated differential genes in RNA sequencing, MS, and IP-MS enriched genes. **C–F** Analysis of SMAD family members (SMAD2, SMAD3, and SMAD4) mRNA relative expression (left panel) and protein levels (right panel) in control and NICD3‑overexpressing CRC cells (*n* = 3). **G, H** Western blot analysis was performed to measure the levels of SMAD3 and TGFBI in control and NICD3-overexpressing CRC cells, with or without SMAD3 knockdown. **I** Representative images of HUVEC tube formation with CM from the control and SMAD3 silencing CRC cells (left panel). Quantitative analysis of the total branching length (*n* = 6, right panel). Scale bar, 50 μm. **J** Representative images of HUVEC migration with CM from the control and SMAD3 silencing CRC cells (left panel). Quantitative analysis of migrated cells (*n* = 5–6, right panel). Scale bar, 50 μm. Data are presented as mean ± SD. Statistical significance was determined by Student’s *t-*test (**C**, **E**) and one-way ANOVA (**I**, **J**). *P < 0.05, **P < 0.01, ***P < 0.001; ns not significant.

Since NICD3 cannot directly function as a transcription factor to regulate TGFBI transcription, we hypothesized that one or more transcription factors mediate the regulatory effect of NICD3 on TGFBI expression. To identify such mediators, we performed MS-based proteomic analysis using NICD3-overexpressing and control HCT116 cells. This analysis identified 1263 upregulated proteins. Furthermore, immunoprecipitation coupled with mass spectrometry (IP-MS) was employed to detect proteins that physically interact with NICD3 under overexpression conditions, yielding 1245 candidate proteins. Intersecting these proteomic results with the upregulated genes from the previous RNA sequencing data identified 12 potential candidates (Fig. 4A, B). Among these, SMAD3 was identified as a key candidate, as it is the canonical transcription factor downstream of TGF-β signaling, a pathway known to exert direct transcriptional control over the TGFBI gene [28].

Building on the above findings implicating SMAD3, we investigated whether NICD3 influences the expression of SMAD family members. We first confirmed that NICD3 overexpression in CRC cells upregulated the mRNA and protein levels of key SMAD family members (SMAD2, SMAD3, and SMAD4), but did not alter SMAD3 phosphorylation (Figs. 4C–F and S3A, B). Under physiological conditions, SMAD2/3 forms a complex with SMAD4 in the cytoplasm, which subsequently translocates to the nucleus to participate in target gene transcription [30]. To investigate whether NICD3 interacts with SMAD family members, we performed co-immunoprecipitation (Co-IP) assay in CRC cell lines overexpressing Flag-tagged NICD3. The results demonstrated that Flag-tagged NICD3 specifically interacts with SMAD3, but not with SMAD2 or SMAD4 (Fig. S3C), consistent with our proteomic analysis.

To determine whether SMAD3 is functionally required for NICD3-driven TGFBI expression and angiogenesis, we performed siRNA-mediated SMAD3 knockdown in NICD3-overexpressing cells (Fig. S3D). SMAD3 silencing effectively reversed NICD3-induced TGFBI upregulation at the protein level (Fig. 4G, H), establishing SMAD3 as a necessary downstream effector. Crucially, SMAD3 knockdown also markedly attenuated the pro-angiogenic capacity of CM, as evidenced by impaired HUVEC tube formation and migration (Fig. 4I-J). This finding was further corroborated using the pharmacological SMAD3 inhibitor SIS3, which similarly suppressed the enhanced tube formation and migratory capacities of HUVECs induced by NICD3-overexpressing CRC cells-derived CM (Fig. S3E, F).

In summary, our data delineate a novel NICD3/SMAD3/TGFBI axis, wherein NICD3 physically interacts with SMAD3 to drive TGFBI expression and subsequent angiogenesis, independently of canonical SMAD3 phosphorylation.

### NICD3 binds to the MH2 domain of SMAD3 to regulate TGFBI gene transcription

**Fig. 5:**
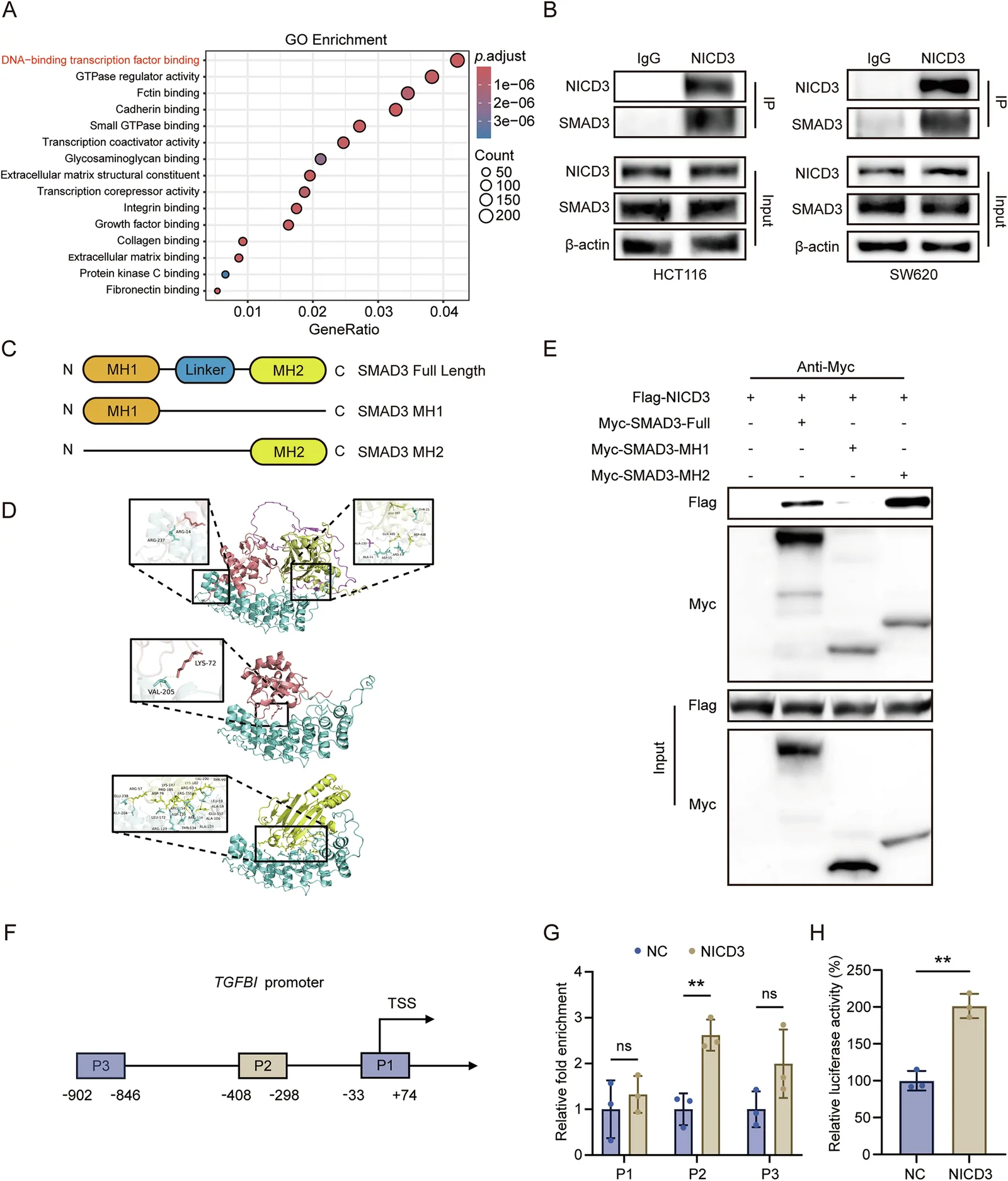
NICD3 directly interacts with the MH2 domain of SMAD3 protein, regulating TGFBI transcription. **A** Gene Ontology enrichment analysis of NOTCH3^high^ and NOTCH3^low^ groups in CRC patients based on the TCGA dataset. **B** Co-IP assay detected the interaction between NICD3 and SMAD3 in NICD3-overexpressing HCT116 and SW620 cells. **C** Schematic representation of SMAD3 structural domains. **D** Structural prediction of the NICD3-SMAD3 complex by AlphaFold3. **E** Exogenous Co-IP assay detected the interaction between Flag-NICD3 and Myc-SMAD3-MH2 in HEK293T cells. **F** Schematic illustration of the *TGFBI* promoter region, indicating the locations of three sets of primers (P1, P2, and P3) relative to the transcription start site. **G** CUT&RUN-qPCR analysis of SMAD3 recruitment at the *TGFBI* promoter (P1-P3) in control and NICD3-overexpressing HCT116 cells. Enrichment was calculated as a fold change relative to the Normal Rabbit IgG control (*n* = 3). **H** Luciferase activity of *TGFBI* promoter in control and NICD3-overexpressing HCT116 cells (*n* = 3). Data are presented as mean ± SD. Statistical significance was determined by Student’s *t-*test (**G**, **H**). **P < 0.01; ns not significant.

Given that NICD3 upregulates TGFBI via SMAD3 without altering its phosphorylation (Fig. S3A, B), we sought to delineate the underlying molecular mechanism. We first interrogated a TCGA cohort stratified by NOTCH3 expression. Gene Ontology enrichment analysis of differentially expressed genes revealed significant enrichment in the “DNA-binding transcription factor binding” term (Fig. 5A), prompting the hypothesis that NICD3 exerts its function through direct protein-protein interactions.

While the canonical Notch3 pathway involves binding to the DNA-binding protein RBPJ to regulate transcription, mounting evidence indicates that NICD3 frequently engages in crosstalk with other signaling pathways, such as Wnt, PI3K-AKT, and TGF-β. Notably, these non-canonical interactions often exert their effects independently of RBPJ, instead interacting directly with effector molecules from other pathways to modulate tumor progression [14, 19]. Guided by this concept, we sought to determine if NICD3 regulates TGFBI through a direct, non-canonical interaction with SMAD3. First, we confirmed the protein-protein interaction between endogenous NICD3 and SMAD3 by Co-IP in NICD3-overexpressing CRC cells (Fig. 5B). To further map the binding region of NICD3 on SMAD3, we analyzed the SMAD3 amino acid sequence, which consists of three domains: the MH1 domain, a linker region, and the MH2 domain (Fig. 5C). We then employed AlphaFold3 to predict the potential binding domain between SMAD3 and NICD3. To enhance prediction accuracy, we removed the intrinsically disordered regions of NICD3 and performed docking simulations with full-length SMAD3, its MH1 domain, and its MH2 domain separately. AlphaFold3 predictions indicated that all three constructs represented potential interaction domains, with notably more predicted binding sites identified between NICD3 and the SMAD3 MH2 domain (Fig. 5D). Experimental validation via exogenous Co-IP in HEK293T cells demonstrated that NICD3 interacts with full-length SMAD3 and exhibits a strong binding preference for the MH2 domain over the MH1 domain (Fig. 5E). This suggests that NICD3 binding to the SMAD3 MH2 domain may enhance the transcriptional activity of SMAD3, thereby facilitating the transcription of downstream target genes.

To elucidate the functional impact of the NICD3-SMAD3 interaction on TGFBI transcription, we performed high-sensitivity CUT&RUN-qPCR analysis in control and NICD3-overexpressing HCT116 cells. By designing three sets of primers (P1, P2, and P3) spanning the *TGFBI* promoter region (Fig. 5F), we observed a significant and localized enrichment of SMAD3 occupancy specifically at the P2 region upon NICD3 overexpression. Although marginal upward trends were observed at P1 and P3, no statistically significant recruitment was detected at these flanking regions, highlighting the localized specificity of SMAD3 binding at the P2 locus (Fig. 5G). This NICD3-dependent recruitment of SMAD3 to the *TGFBI* promoter was functionally corroborated by a robust increase in *TGFBI* promoter-driven luciferase activity (Fig. 5H).

In summary, these results elucidate a mechanism whereby NICD3 directly binds to the MH2 domain of SMAD3, thereby facilitating its targeted recruitment to the *TGFBI* promoter and driving its transcription to promote tumor angiogenesis.

### HES targets TGFBI to suppress CRC progression via anti-angiogenesis

Having elucidated the mechanism of the non-canonical NICD3/TGFBI signaling cascade, we next evaluated its therapeutic targetability for the pro-angiogenic CRC subtype. Given that the clinical development of direct NOTCH inhibitors is often hindered by significant gastrointestinal toxicity [31, 32], targeting downstream effector TGFBI represents a promising alternative approach. Existing studies have identified TGFBI as a potential predictor of anti-tumor immune signatures [33], suggesting broader translational prospects for TGFBI targeting. Molecular docking serves as a preliminary screening method to rapidly identify potential binding compounds and modes. We modeled the TGFBI protein structure and performed molecular docking, which identified nine candidate small-molecule compounds with absolute docking scores above 13 (Table S3). The top-ranked compound, 1,3,6-Tri-O-galloyl-beta-D-glucose (1,3,6-TGG; IC₅₀ = 305.2 μM), significantly inhibited HUVEC tube formation but failed to suppress migration (Fig. S4A–D). A subsequent screen of Eriocitrin (ERI), a compound with reported anticancer properties [34], showed neither cytotoxic effect on HCT116 cells nor inhibition of HUVEC tube formation or migration (Fig. S4E–H).

**Fig. 6:**
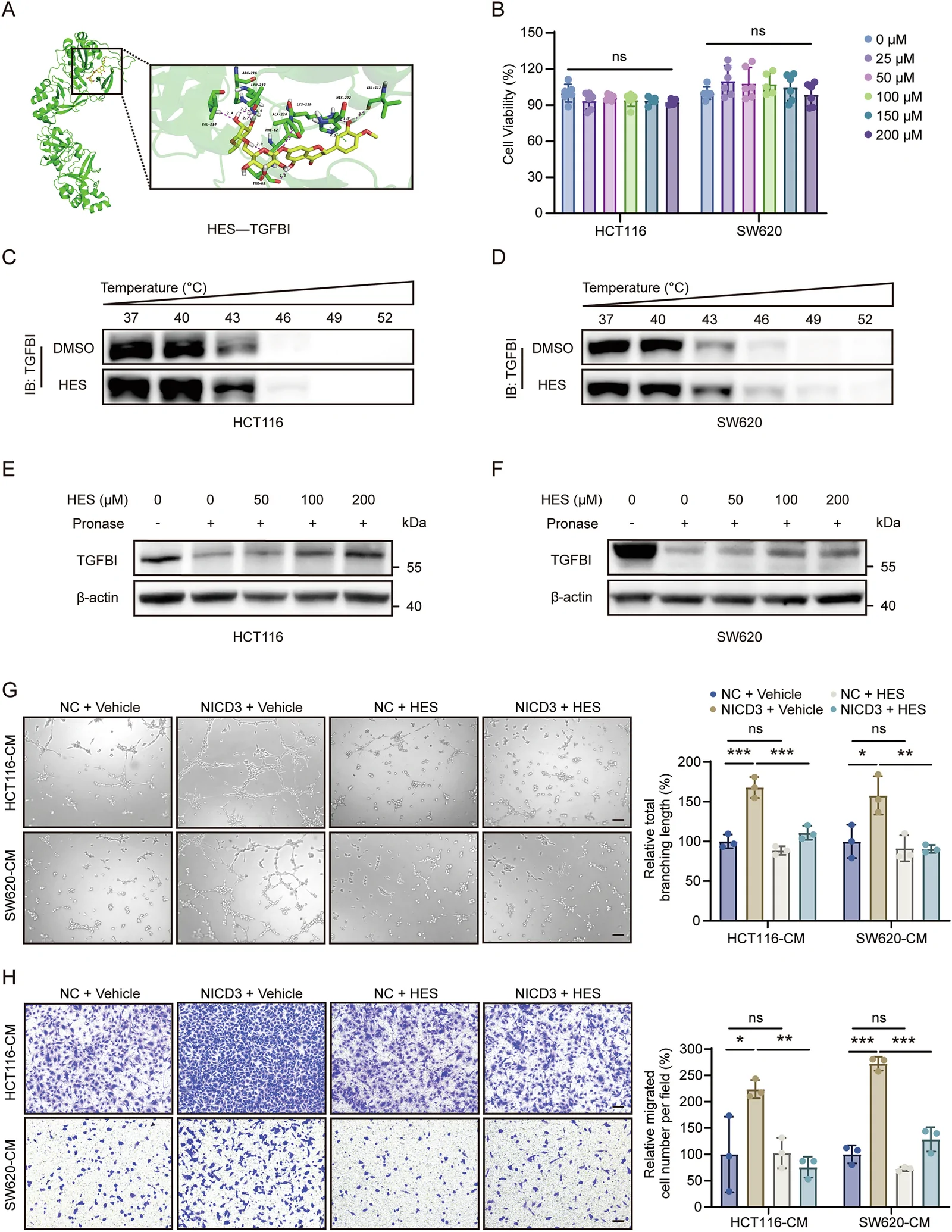
HES directly targets TGFBI to neutralize NICD3-induced pro-angiogenic effects in vitro. **A** The molecular docking model of HES and TGFBI. **B** The bar graph presented the cell viability of HCT116 and SW620 cells under different HES concentrations (*n* = 4–6). **C, D** The CETSA assay characterized the thermal stabilization of TGFBI by HES in NICD3-overexpressing HCT116 and SW620 cells. **E, F** The DARTS assay evaluated the binding of HES to TGFBI based on proteolytic resistance in NICD3-overexpressing HCT116 and SW620 cells. **G** Representative images of HUVEC tube formation with CM from control and NICD3-overexpressing CRC cells, treated with either vehicle or HES (200 μM) (left panel). Quantitative analysis of the total branching length (*n* = 3, right panel). Scale bar, 50 μm. **H** Representative images of HUVEC migration with CM from control and NICD3-overexpressing CRC cells, treated with either vehicle or HES (200 μM) (left panel). Quantitative analysis of migrated cells (*n* = 3, right panel). Scale bar, 50 μm. Data are presented as mean ± SD. Statistical significance was determined by one-way ANOVA (**B**, **G**, **H**). *P < 0.05, **P < 0.01, ***P < 0.001; ns not significant.

We therefore focused on HES, a flavonoid compound derived from citrus peel extracts. Numerous studies have reported its significant biological protective functions. For instance, HES protects cardiovascular health by improving blood pressure and lipid parameters, provides neuroprotection by alleviating oxidative stress and neuroinflammation, and modulates metabolic dysfunction in diabetes, obesity, and liver diseases [35–37]. Previous studies have demonstrated that HES inhibits the proliferation of various cancer cell types, including breast, cervical, and lung cancers [38–40], and one report indicated it suppresses CRC growth via the glycolytic pathway [41]. Molecular docking predicted stable binding between HES and TGFBI (Fig. 6A). To evaluate the therapeutic potential of HES, we first examined its direct effects on CRC cell viability. HES treatment exhibited negligible intrinsic cytotoxicity and failed to significantly inhibit the proliferation of NICD3-overexpressing CRC cells in vitro (Fig. 6B). Crucially, the direct interaction was validated through both thermal and biochemical stability assays. The CETSA assay demonstrated that HES significantly enhanced the thermal stability of TGFBI in CRC cells (Figs. 6C, D and S4I). Furthermore, the DARTS assay revealed that TGFBI became increasingly resistant to protease digestion in the presence of HES in a dose-dependent manner (Fig. 6E, F), further corroborating the robust physical interaction between HES and TGFBI. Functionally, HES treatment effectively neutralized the pro-angiogenic capacity of CM from NICD3-overexpressing cells (Fig. 6G, H), while exhibiting no direct effect on HUVECs (Fig. S4J).

**Fig. 7:**
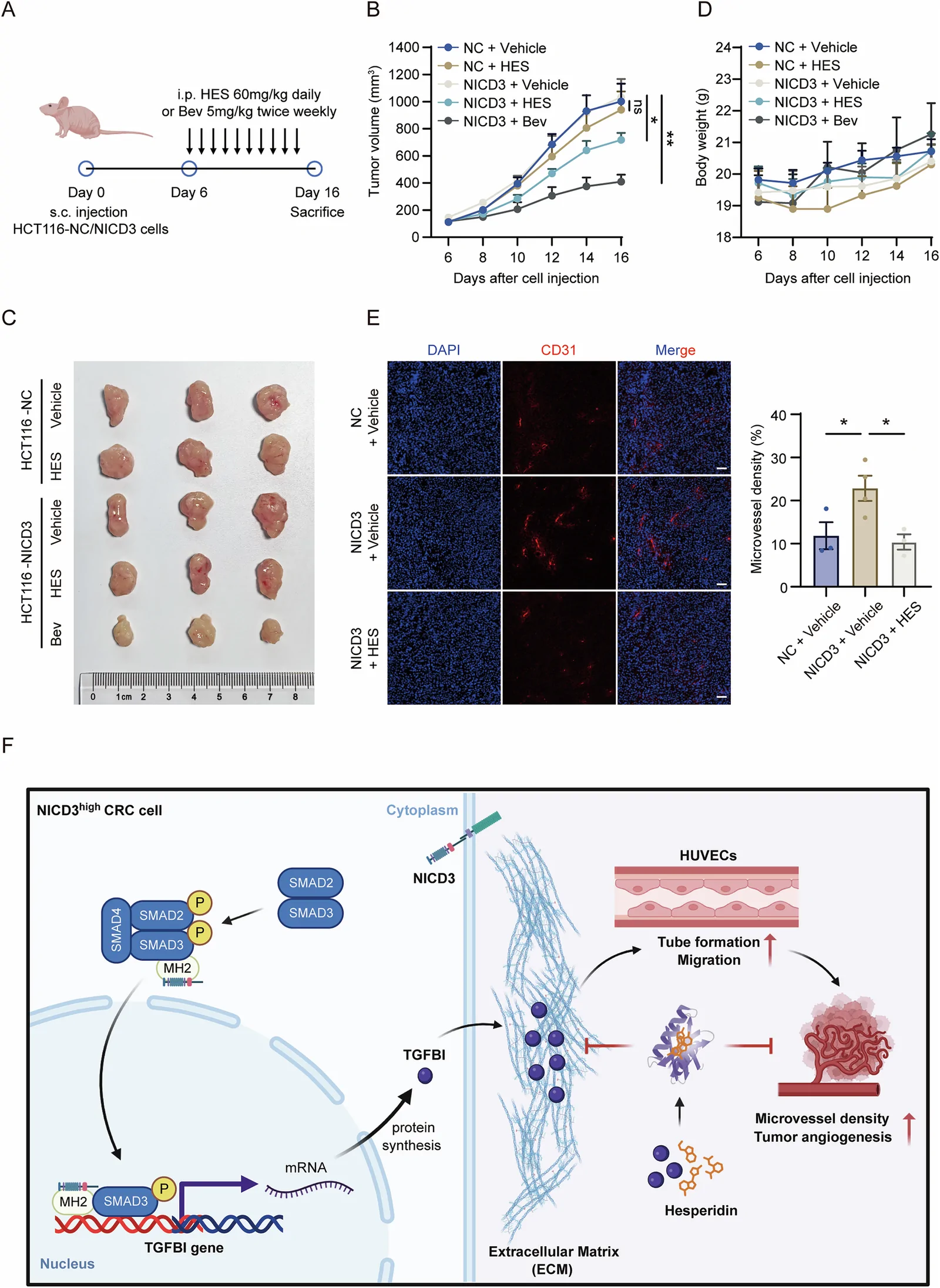
HES suppresses NICD3-driven CRC progression and angiogenesis. **A** Schematic of the experimental timeline. Nude mice were subcutaneously inoculated with HCT116-NC or HCT116-NICD3 cells, followed by HES or Bev treatment starting at day 6. **B** Tumor growth curves of HCT116-NC and HCT116-NICD3 xenografts following treatment with HES (60 mg/kg), Bev (5 mg/kg), or vehicle (*n* = 4–5). **C** Representative images of subcutaneous xenografts from the indicated groups at the end of the experiment. **D** Changes in body weight of mice throughout the treatment period (*n* = 4–5). **E** Representative immunofluorescence images (left panel) and quantification (*n* = 3, right panel) of CD31 expression in the key mechanistic cohorts of HCT116 xenografts. Scale bar, 50 μm. **F** Mechanism overview of a non-canonical NICD3/SMAD3/TGFBI axis driving CRC angiogenesis and its therapeutic target by HES. Data are presented as mean ± SD. Statistical significance was determined by two-way ANOVA (**B**) and one-way ANOVA (**E**). *P < 0.05, **P < 0.01; ns not significant.

To evaluate the in vivo therapeutic efficacy of HES, we established subcutaneous xenograft models in nude mice (Fig. 7A). HES selectively suppressed the growth of NICD3-overexpressing tumors without affecting NC tumors, confirming its high target specificity. With the clinical anti-angiogenic agent Bev serving as a positive control, HES exhibited a robust and significant reduction in tumor volume compared to the NICD3 vehicle group, although its potency was slightly lower than that of the clinical benchmark (Fig. 7B, C). Furthermore, no significant body weight loss was observed in HES-treated mice, indicating a favorable safety profile (Fig. 7D).

To elucidate the underlying anti-angiogenic mechanism, we focused on the key mechanistic cohorts (Fig. S5A, B). Consistent with our in vitro findings, immunofluorescence analysis revealed that HES treatment markedly attenuated MVD (CD31^+^) in NICD3-overexpressing tumors (Fig. 7E), validating its potent anti-angiogenic efficacy within the tumor microenvironment. Histological assessment of major organs further corroborated the favorable safety profile of HES, with no structural or pathological damage observed (Fig. S5C).

In summary, these findings demonstrate that HES is a potent TGFBI-targeting agent that inhibits NICD3-induced CRC progression by disrupting the angiogenic niche without systemic toxicity.

## Discussion

In this study, we investigated a critical signaling axis that may sustain the aggressive vascular phenotype of CRC, a key feature whose molecular drivers remain to be fully elucidated. Our study suggests that NICD3 may regulate this process via the transcriptional upregulation of TGFBI. This mechanism is particularly noteworthy given our observation that NICD3 overexpression unexpectedly attenuated CRC cell proliferation in vitro, yet modestly promoted tumor growth in vivo. This discrepancy strongly suggests that NICD3-driven CRC progression is not a cell-autonomous proliferative effect but is instead critically dependent on the remodeling of the tumor microenvironment. Specifically, NICD3 appears to “switch” the tumor cell’s priority toward secretome modulation. Our proteomic profiling of the secretome, combined with the finding that rTGFBI alone can mimic NICD3-induced tube formation and migration whereas TGFBI silencing attenuated this effect, identifies TGFBI as the indispensable executor of this angiogenic program. Further mechanistic dissection of NICD3-induced TGFBI upregulation revealed a non-canonical ligand-independent NICD3-SMAD3 interaction. Unlike the classical model where Notch amplifies TGF-β ligand secretion to activate SMADs, our data indicate that NICD3 can directly recruit SMAD3 to chromatin to initiate TGFBI transcription. This mechanism appears to bypass upstream receptor activation steps, providing a plausible molecular explanation for the unique angiogenic features observed in NICD3^high^ CRC.

Our findings offer a potential explanation for a longstanding clinical paradox in CRC therapy: specifically, while CMS4 tumors exhibit hyperactivated TGF-β signaling [6], therapies targeting TGF-β receptors have demonstrated limited efficacy in clinical trials [42, 43]. Canonical Notch-TGF-β crosstalk typically relies on ligand-receptor engagement [44]. However, we observed a “short-circuit” mechanism wherein NICD3 binds to the MH2 domain of SMAD3 to drive the expression of pro-angiogenic genes, such as TGFBI, without requiring canonical receptor activation. This ligand-independent characteristic may explain why TGF-β ligand traps or receptor kinase inhibitors frequently fail in this subtype, as the angiogenic signal is intrinsically sustained by high NICD3 levels. Consequently, targeting the NICD3-SMAD3 interface or its downstream effector, TGFBI, might represent a more promising therapeutic strategy.

Notably, Direct inhibition of NOTCH [31, 32] or global blockade of SMAD3 [45] is often limited by potential toxicities or disruption of tissue homeostasis. In this context, identifying TGFBI as a key effector holds potential translational significance [33, 46]. Currently, although VEGF remains the primary target for anti-angiogenic therapy (e.g., Bev) [11, 47], tumor vessels often develop resistance via compensatory pathways. TGFBI, functioning as an extracellular matrix protein, is known to promote endothelial migration by engaging integrins [28, 48], thereby providing an alternative pathway to circumvent VEGF blockade [49].

To explore clinical translation, we propose HES as a candidate compound targeting the NICD3-TGFBI axis. Our results demonstrate that HES specifically targets TGFBI to suppress NICD3-driven angiogenesis, offering a potential strategy for patients refractory to standard anti-VEGF regimens. The robust physical interaction between HES and TGFBI was rigorously validated through both CETSA and DARTS assays, confirming that HES enhances TGFBI stability and confers resistance to proteolysis. Through functional validation, we found that HES specifically inhibits pro-angiogenic activity. Unlike direct NOTCH inhibitors that cause gastrointestinal toxicity, HES exhibits a favorable safety profile [41, 50–52], confirmed by H&E histological assessment of major organs and stable body weight. This suggests that utilizing HES to promote tumor vessel normalization could be a promising strategy to complement existing cytotoxic therapies.

Beyond the direct binding of HES to TGFBI to inhibit pro-angiogenic activity, the therapeutic potential of HES may be amplified by its ability to modulate the gut-tumor microbial ecosystem, a factor increasingly recognized as a determinant of CMS4 development [53–55]. CMS4 tumors are often enriched with “oncomicrobes” like *Bacteroides fragilis* (ETBF) and *Fusobacterium animalis*, which drive EMT and stromal remodeling [56, 57]. Emerging evidence suggests that HES can restore the intestinal barrier and enrich SCFA-producing bacteria [57, 58], which may effectively antagonize the immunosuppressive and pro-angiogenic signals produced by these CMS4-specific pathogens [59, 60]. This suggests that HES may act as a multimodal agent—silencing the NICD3 axis while simultaneously reshaping the microbial landscape to trigger systemic antitumor immunity [61–63].

Despite these insights, this study has certain limitations. First, while efficacy was confirmed in cell-derived models, rigorous pharmacokinetic analyses and validation in patient-derived xenograft models are required to establish in vivo utility. Furthermore, although we established TGFBI as a key pro-angiogenic molecule in CRC, the specific downstream receptors and signaling pathways mediating its pro-angiogenic effects remain incompletely elucidated and warrant further investigation. In addition, the in vivo efficacy and pharmacological profile of HES, despite its promising translational potential, need to be more thoroughly characterized. Collectively, our findings uncover a non-canonical NICD3/SMAD3 pathway that drives angiogenesis in CRC by directly activating TGFBI transcription. Mechanistically, high levels of NICD3 interact with the SMAD3 MH2 domain to form a transcriptional complex that bypasses TGF-β pathway requirement, thereby elucidating the unclear regulatory mechanism of TGFBI. Furthermore, we identify the natural compound HES as an effective blocker of this pathway, confirming the translational potential of targeting the NICD3/SMAD3/TGFBI axis for anti-angiogenic therapy (Fig. 7F).

## Supplementary information

Supplementary information

Table S2

Original western blots

## Data Availability

The datasets used or analyzed during the current study are available from the corresponding author upon reasonable request.
