# Supplementary material for: NICD3 mediates pro-angiogenic effects through SMAD3/TGFBI axis in colorectal cancer
Source: Cell Death Dis. 2026 Jun 15;17(1):746. doi: 10.1038/s41419-026-08939-z (PMC13493961; doi:10.1038/s41419-026-08939-z)

Original western blots & gels

NICD3 Mediates Pro-angiogenic Effects Through SMAD3/TGFBI Axis in Colorectal Cancer

Fig. 3D HCT116

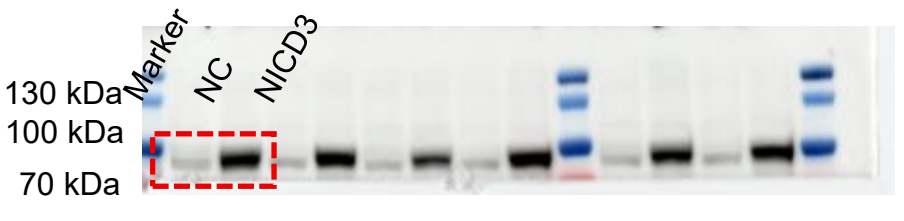

NICD3

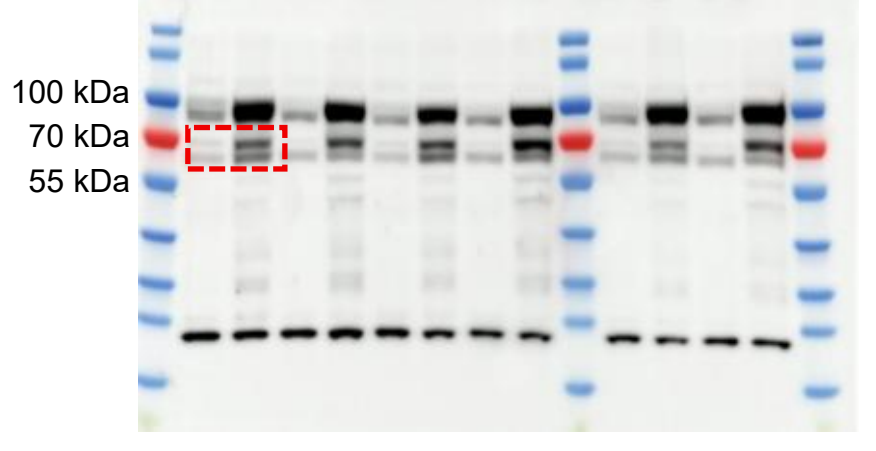

TGFBI

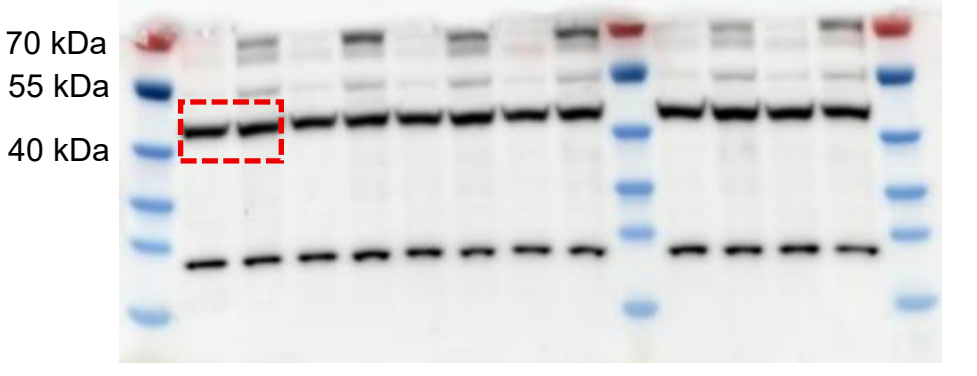

β-actin

Fig. 3D SW620

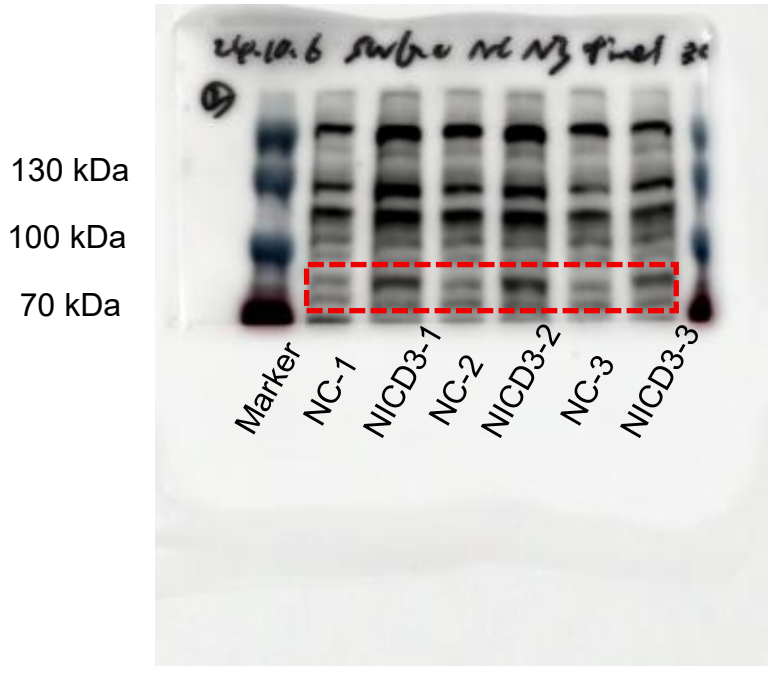

NICD3

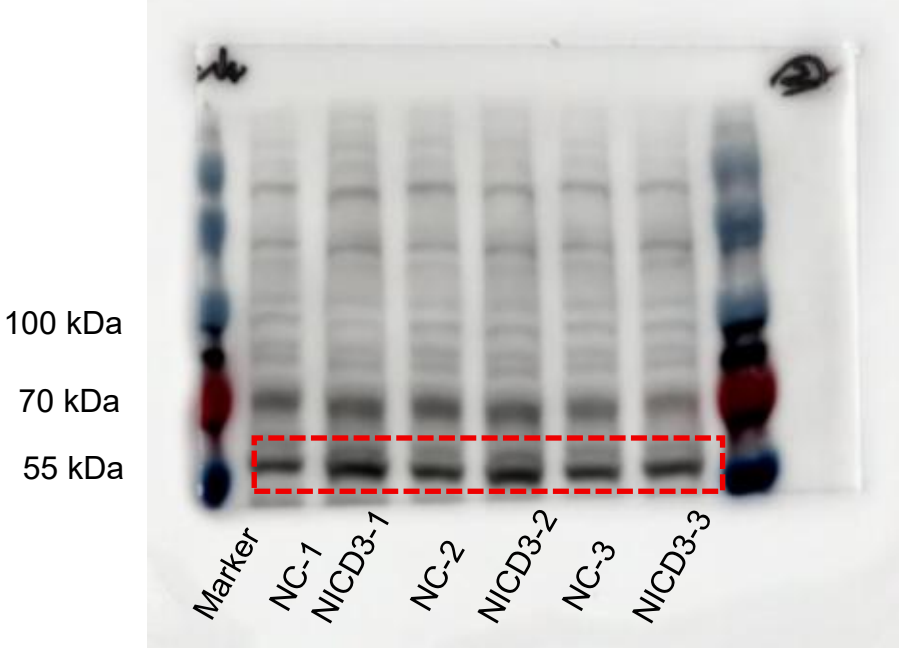

TGFBI

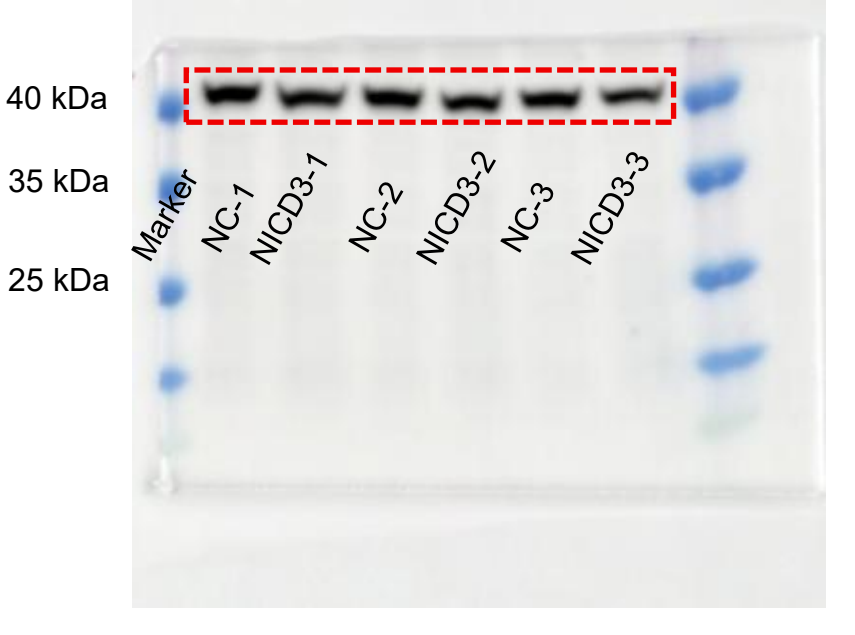

β-actin

Fig. 4D HCT116 SMADs

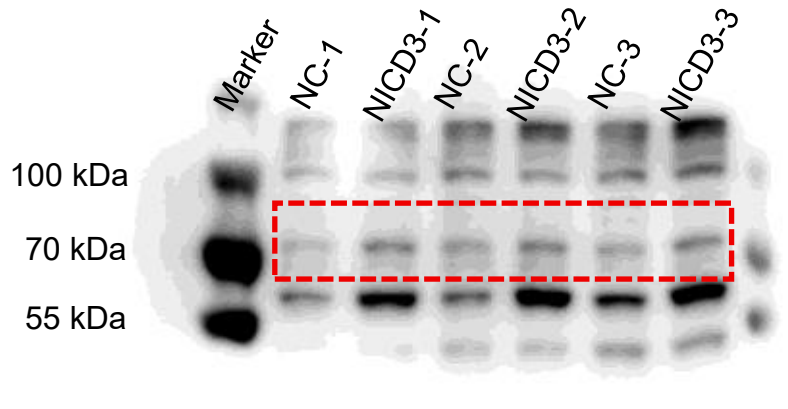

NICD3

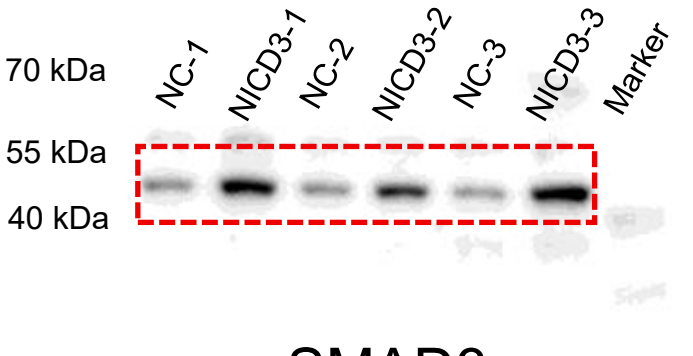

p-SMAD3

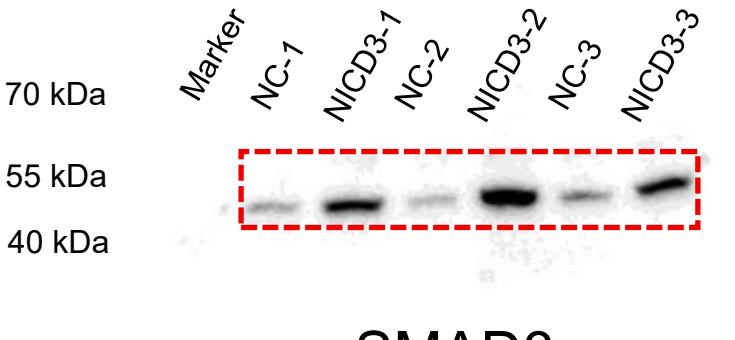

SMAD3

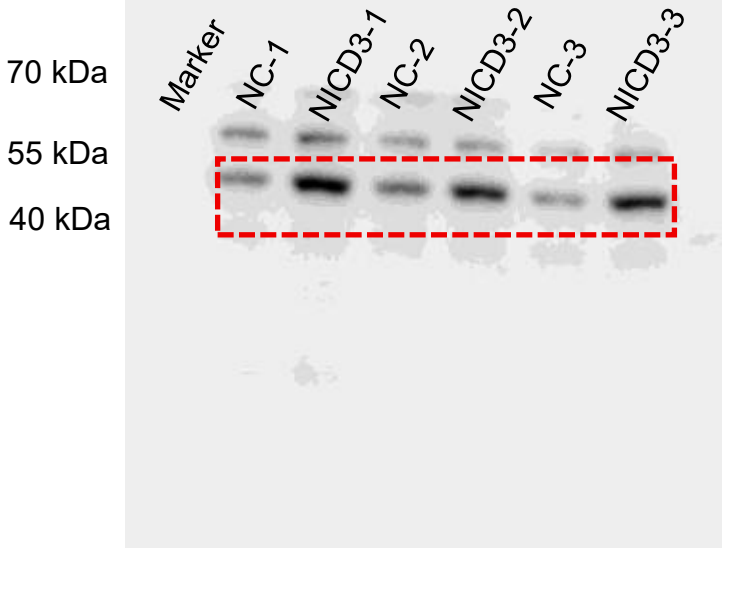

p-SMAD2

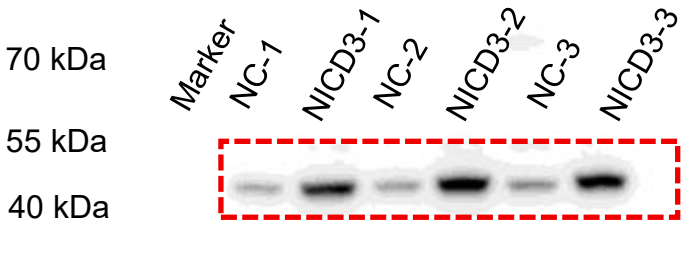

SMAD2

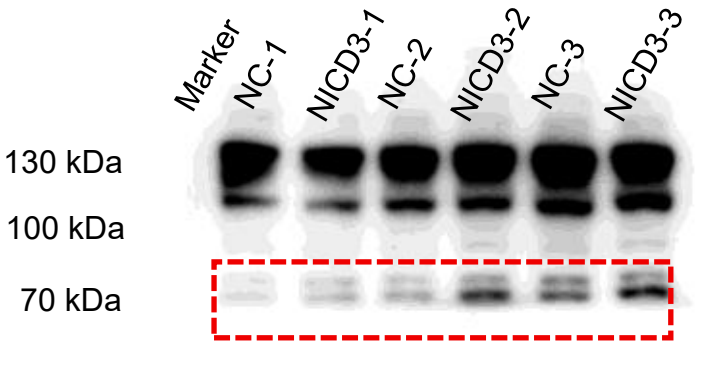

SMAD4

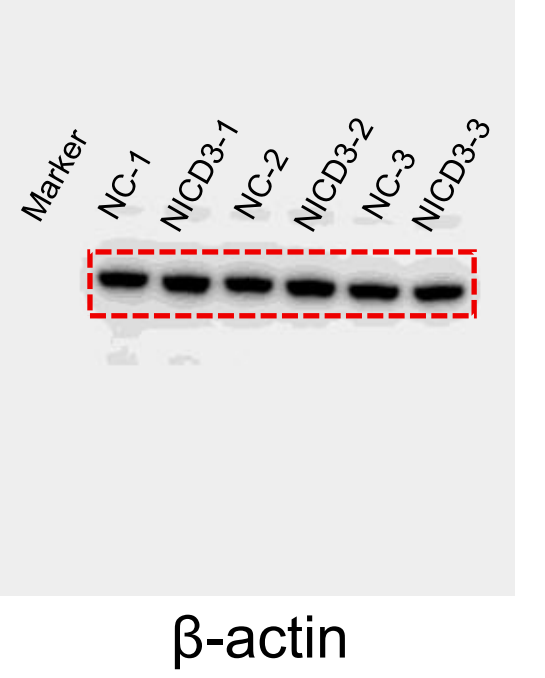

β-actin

Fig. 4F SW620 SMADs

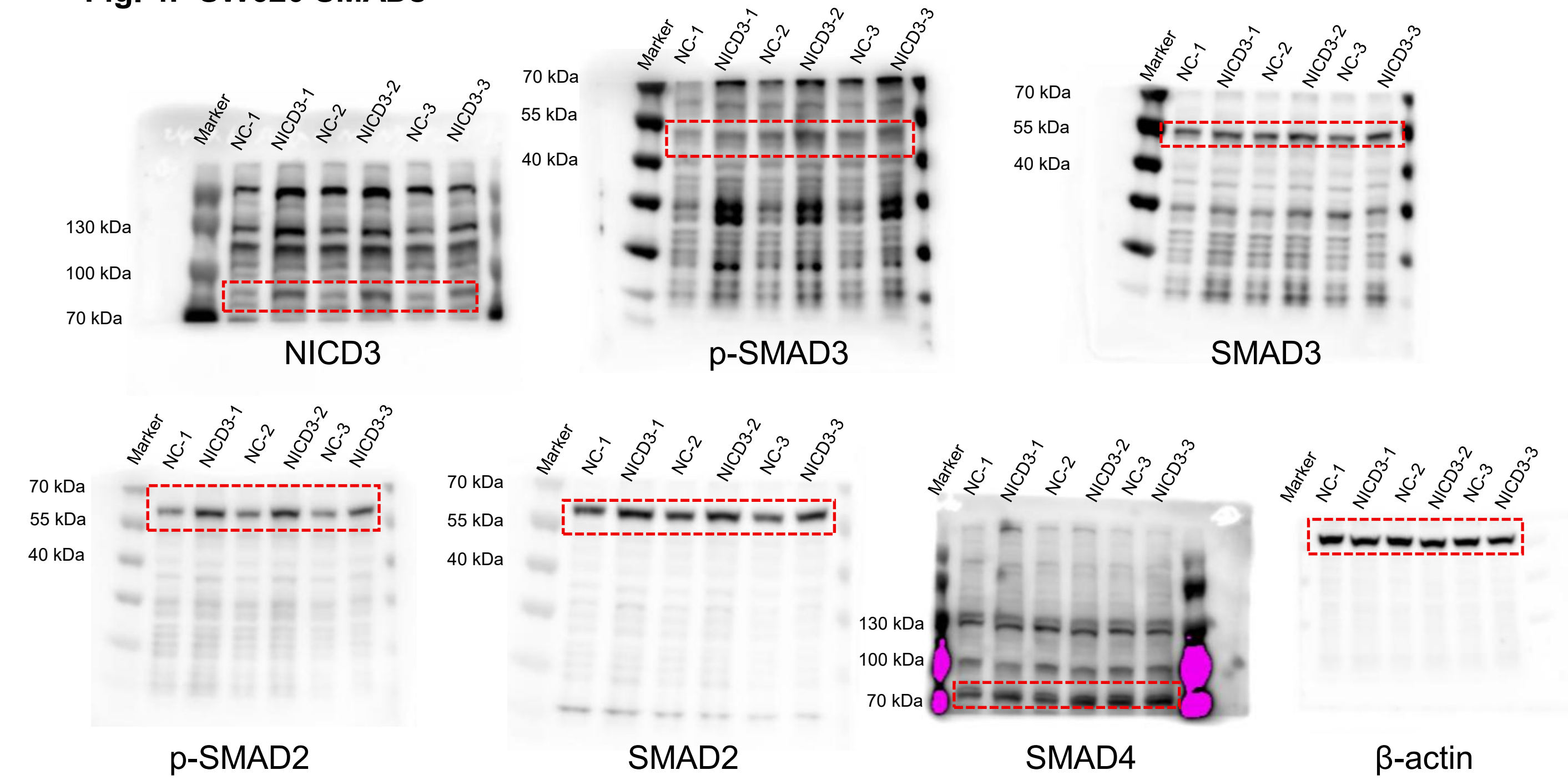

Fig. 4G HCT116 si-SMAD3

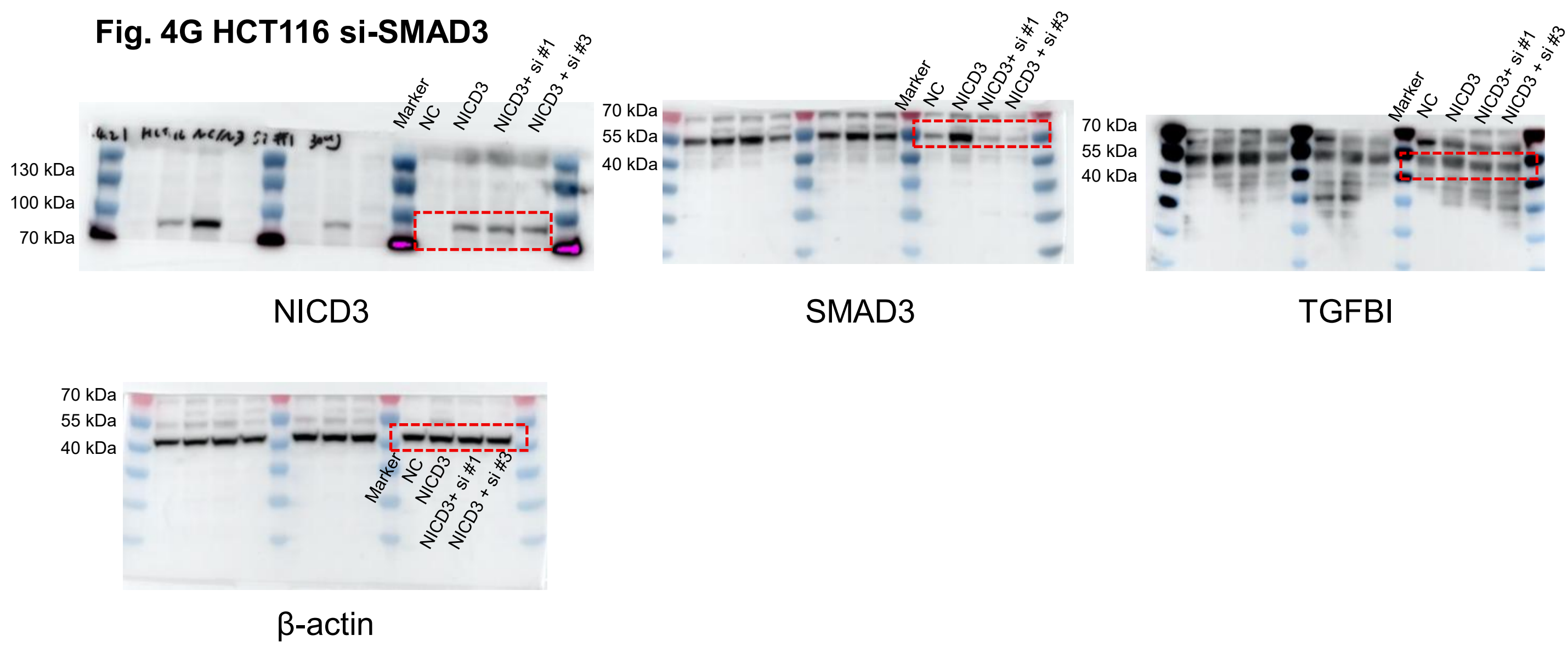

Fig. 4H SW620 si-SMAD3

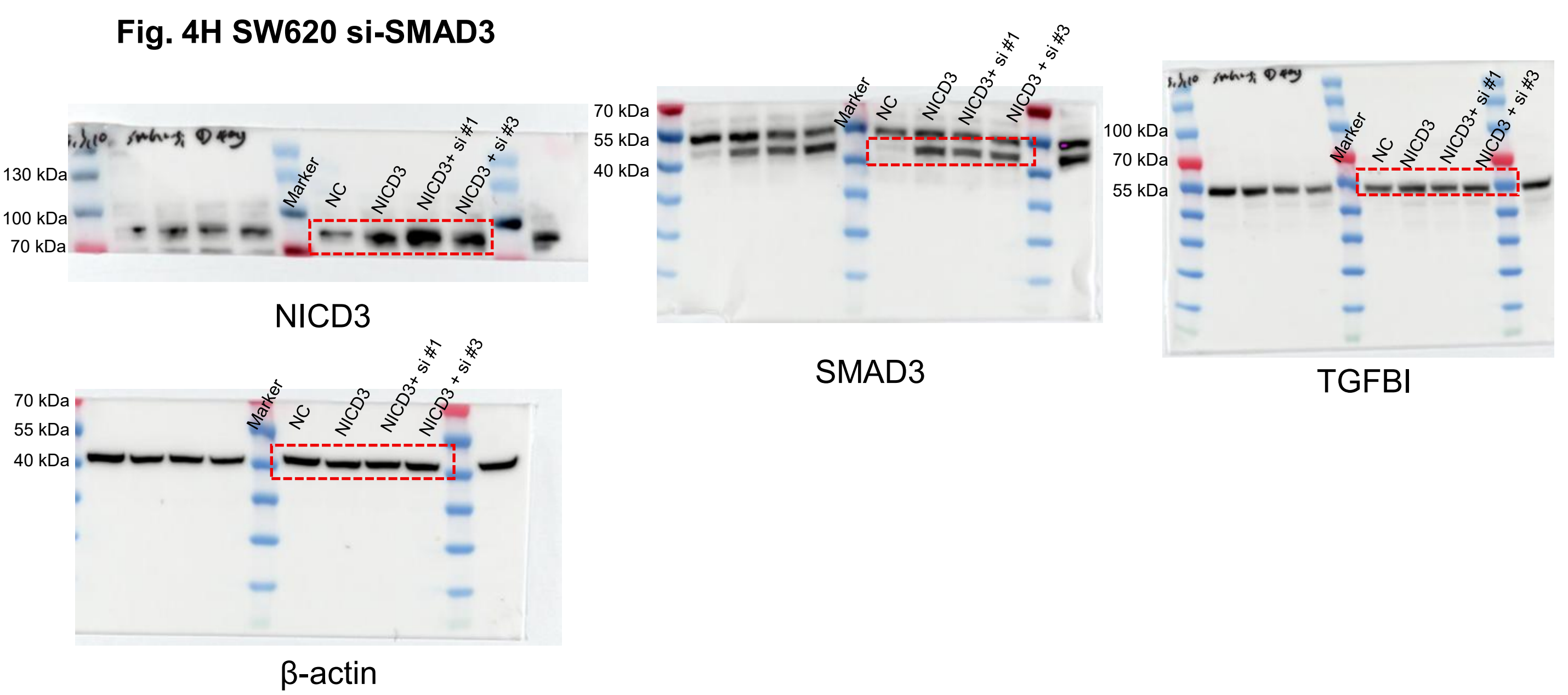

Fig. 5B HCT116 endogenous COIP

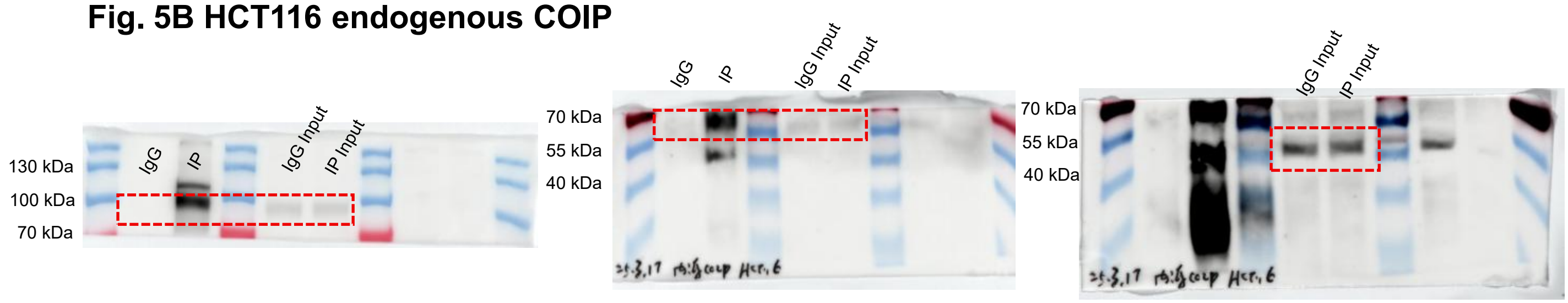

NICD3 SMAD3 β-actin

Fig. 5B SW620 endogenous COIP

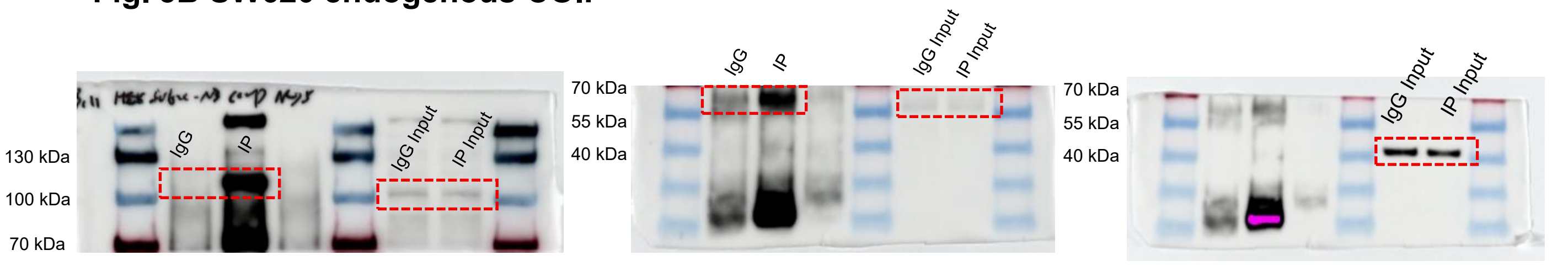

NICD3 SMAD3 β-actin

Fig. 5E exogenous COIP

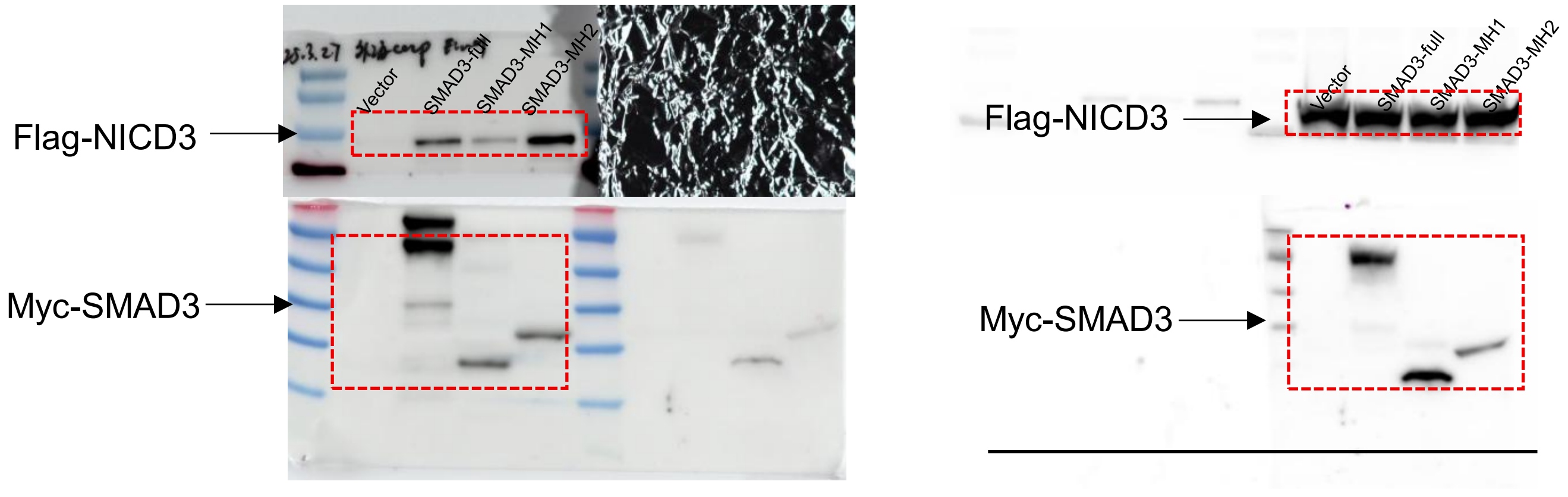

Input

Fig. 6C HCT116 CETSA

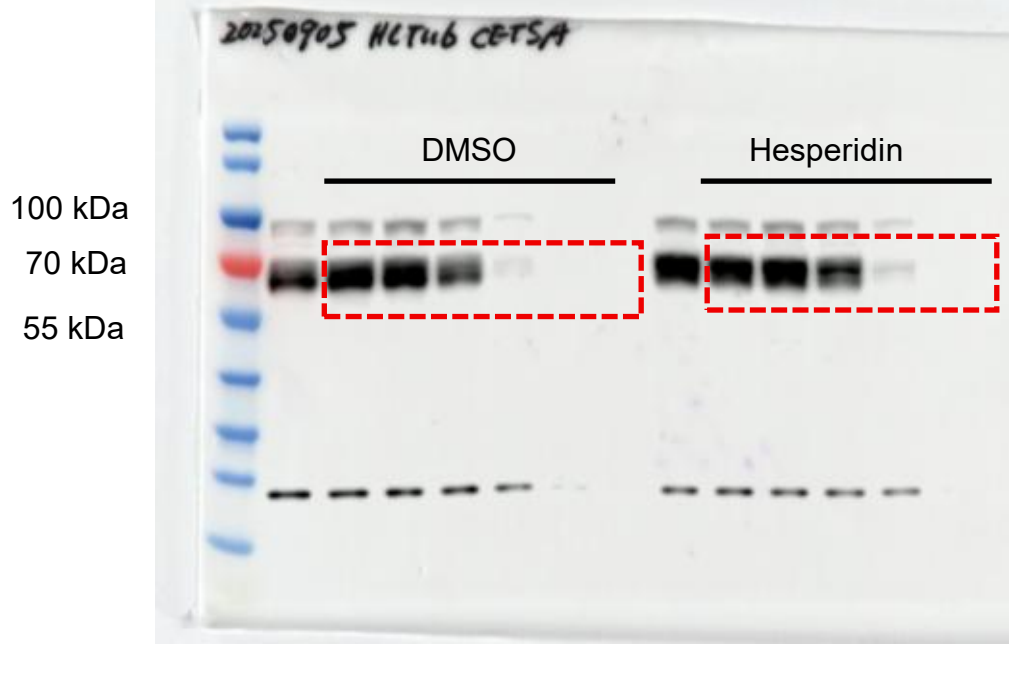

TGFBI

Fig. 6D SW620 CETSA

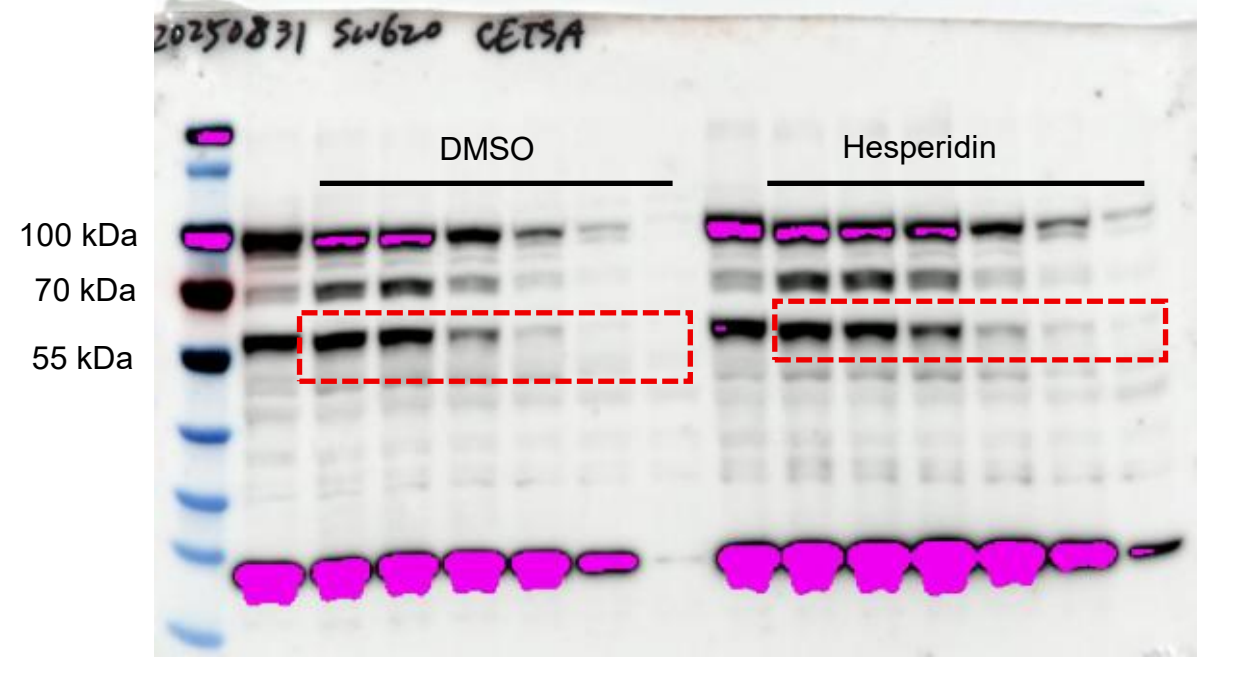

TGFBI

Fig. 6E HCT116 DARTS

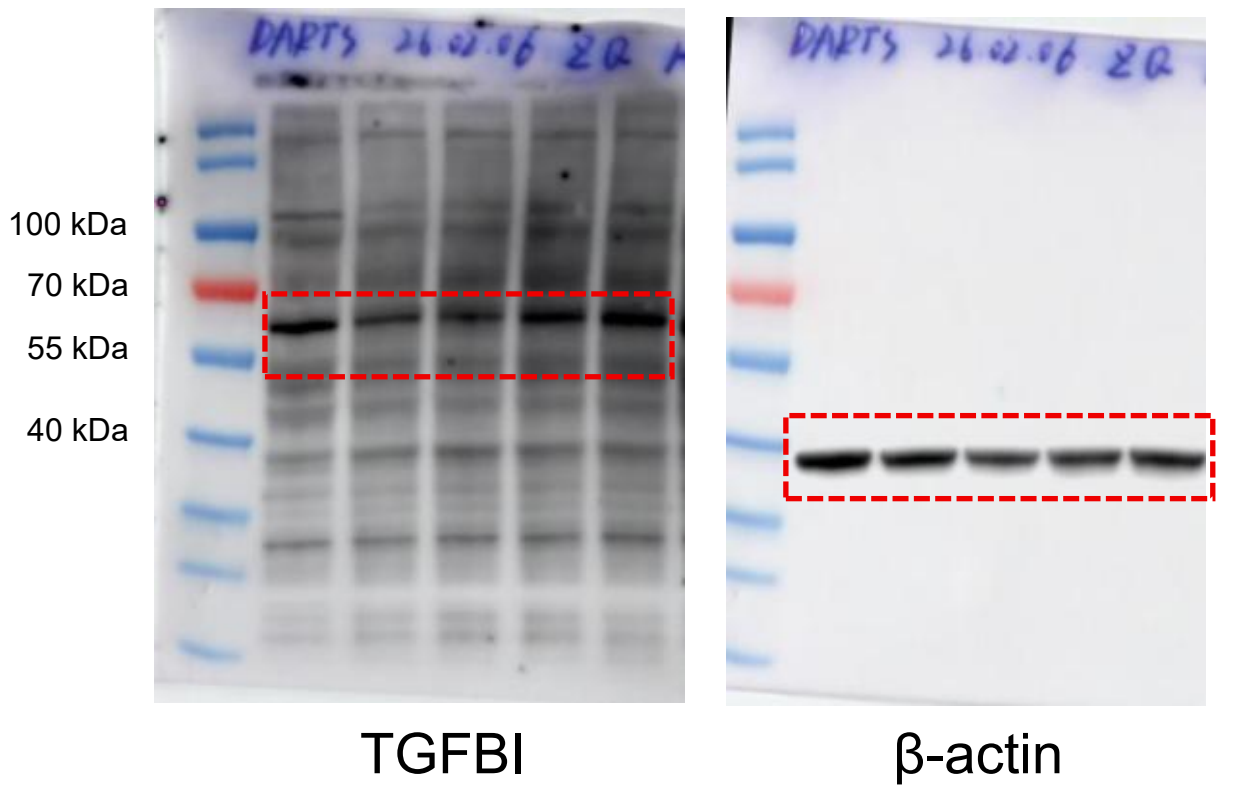

Fig. 6F SW620 DARTS

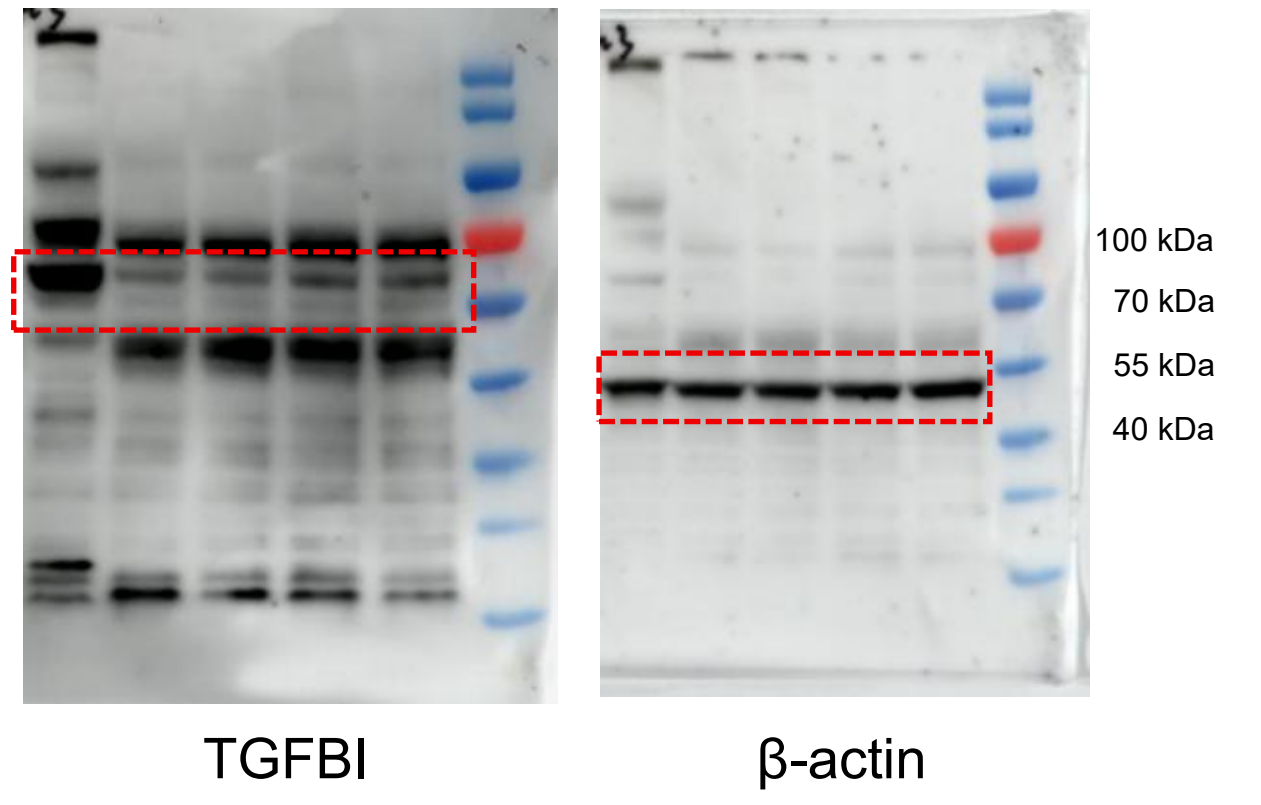

Fig. S2I HCT116-si-TGFBI

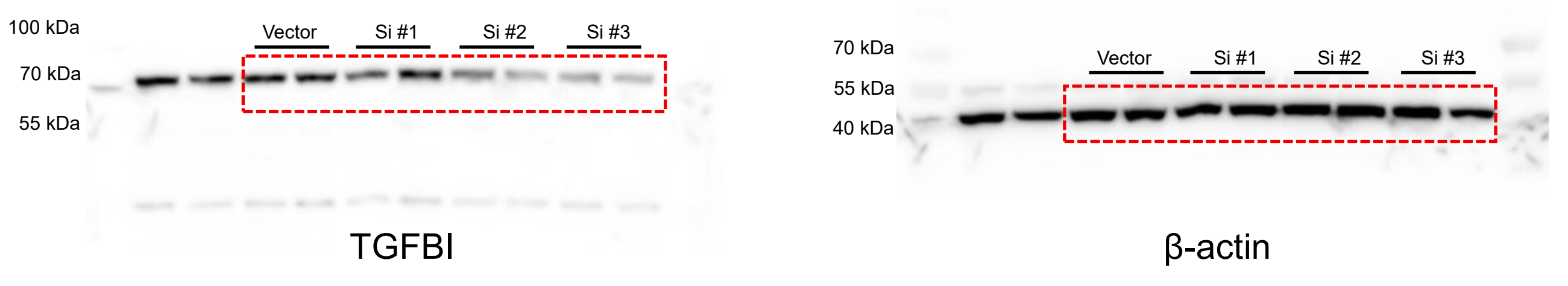

Fig. S3C HCT116 Flag-tag COIP

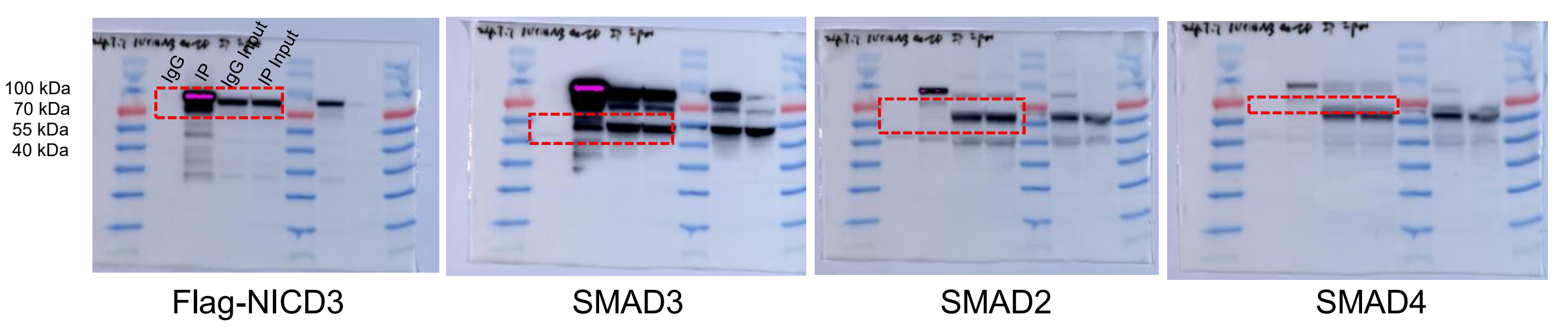

Fig. S3C SW620 Flag-tag COIP

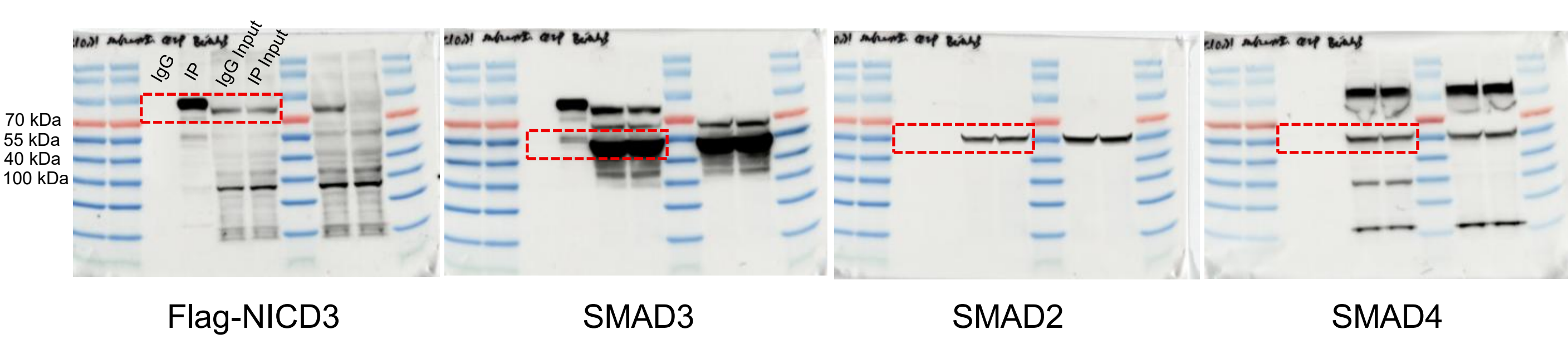

Supplement: Supplementary file 1 [file 41419_2026_8939_MOESM3_ESM.pdf]
