# Supplementary material for: NICD3 mediates pro-angiogenic effects through SMAD3/TGFBI axis in colorectal cancer
Source: Cell Death Dis. 2026 Jun 15;17(1):746. doi: 10.1038/s41419-026-08939-z (PMC13493961; doi:10.1038/s41419-026-08939-z)
Supplement: Supplementary file 2 [file 41419_2026_8939_MOESM1_ESM.docx]

**Supplementary Information**

**NICD3 Mediates Pro-angiogenic Effects Through SMAD3/TGFBI Axis in Colorectal Cancer**

**Supplementary Figures**

**Fig. S1. CRC CMS4 subtype and NOTCH3^high^ patients were significantly enriched in angiogenesis in the GEO dataset. (A)** GSVA and **(B)** GSEA enrichment analysis of CMS4 and CMS others in CRC patients from the GSE39582 dataset. **(C)** GSVA and **(D)** GSEA enrichment analysis of NOTCH3^high^ and NOTCH3^low^ groups in CRC patients from the GSE39582 dataset.

**Fig. S2. Gain of NICD3 function drives CRC angiogenesis, associated with TGFBI. (A-B)** Tumor growth curves of **(A)** HCT116 and **(B)** SW620 xenografts in the control and NICD3-overexpressing groups (*n* = 7–8). **(C-D)** Quantification of HCT116 and SW620 cell proliferative capacity monitored by CCK-8 assay (*n* = 4-6). **(E)** Representative immunofluorescence images (left panel) and quantification of MVD in MC38-NC and MC38-Nicd3 xenografts (*n* = 4, right panel). Scale bar, 50 μm. **(F)** Analysis of the TCGA cohort revealed elevated TGFBI expression in CMS4 and NOTCH3^high^ CRC patients. **(G)** Analysis of the GSE39582 cohort revealed elevated TGFBI expression in CMS4 and NOTCH3^high^ CRC patients. **(H)** Relative protein level of TGFBI in the CM from HCT116 cells stably overexpressing NICD3 or control, as quantified by 5D label-free MS (*n* = 3). **(I)** Western blot analysis exhibited the effective knockdown of TGFBI in HCT116 cells by transfection with si-TGFBI #2 and si-TGFBI #3.

Data are presented as mean ± SD. Statistical significance was determined by Student’s *t* test (E, F, G and H) and two-way ANOVA (A, B, C and D). **P* < 0.05, ****P* < 0.001; ns, not significant.

**Fig. S3. NICD3 promotes angiogenesis by upregulating SMAD3 expression and engaging in a functional interaction in CRC. (A-B)** Western blot quantitative analysis of p-SMAD3/SMAD3 and p-SMAD2/SMAD2 protein expression ratio in control and NICD3-overexpressing **(A)** HCT116 and **(B)** SW620 cells (*n* = 3-6). **(C)** Co-IP assays detected the interaction between Flag-tagged NICD3 and SMAD3 instead of SMAD2 or SMAD4 in NICD3-overexpressing HCT116 and SW620 cells. **(D)** RT-qPCR analysis exhibited the effective knockdown of SMAD3 in HCT116 cells by transfection with si-SMAD3 #1 and si-SMAD3 #3 (*n* = 5). **(E)** Representative images of HUVEC tube formation with CM from the vehicle and SIS3-treated CRC cells (left panel). Quantitative analysis of the total branching length (*n* = 5-6, right panel). Scale bar, 50 μm. **(F)** Representative images of HUVEC migration with CM from vehicle and SIS3-treated CRC cells (left panel). Quantitative analysis of migrated cells (*n* = 6, right panel). Scale bar, 50 μm.

Data are presented as mean ± SD. Statistical significance was determined by Student’s *t* test (A, B) and one-way ANOVA (D, E and F). **P* < 0.05, ***P* < 0.01, ****P* < 0.001; ns, not significant.

**Fig. S4.** **The effects of putative TGFBI-binding compounds 1,3,6-TGG and ERI on NICD3-driven angiogenesis. (A)** The molecular docking binding-mode diagram of 1,3,6-TGG with TGFBI. **(B)** Cell viability of HCT116 cells treated with different concentrations of 1,3,6-TGG (*n* = 5). **(C-D)** Quantitative analysis of HUVEC tube formation and migration with CM from control and NICD3-overexpressing HCT116 cells, treated with either vehicle or 1,3,6-TGG (150 μM) (*n* = 3). **(E)** The molecular docking binding-mode diagram of ERI with TGFBI. **(F)** Cell viability of HCT116 cells treated with different concentrations of ERI (*n* = 4-5). (**G-H**) Quantitative analysis of HUVEC tube formation and migration with CM from control and NICD3-overexpressing HCT116 cells, treated with either vehicle or ERI (200 μM) (*n* = 3-6). **(I)** Quantification of TGFBI thermal stability based on relative protein intensity in NICD3-overexpressing HCT116 and SW620 cells. The relative protein intensity was normalized to the levels at 37°C (*n* = 4-5). (**J**) Cell viability of HUVECs treated with different concentrations of HES (*n* = 4).

Data are presented as mean ± SD, except for (I) which is presented as mean ± SEM. Statistical significance was determined by one-way ANOVA (B, C, D, F, G, H and J) and paired Student’s *t* test (I). **P*< 0.05, ***P*< 0.01, ****P*< 0.001; ns, not significant.

**Fig. S5. HES suppresses NICD3-driven CRC progression with a favorable systemic safety profile in vivo.**

**(A)** Tumor growth curves of HCT116-NC and HCT116-NICD3 xenografts following treatment with HES (60 mg/kg) or vehicle (*n* = 3). **(B)** Representative images of subcutaneous xenografts from mice in the indicated groups. **(C)** Representative H&E staining images of major organs (heart, liver, spleen, lung, and kidney) harvested from the indicated groups at the study endpoint. Scale bar, 100 μm.

Data are presented as mean ± SD. Statistical significance was determined by two-way ANOVA (A). ***P*< 0.01 vs. NICD3 + Vehicle.

**Supplementary Tables**

**Table S1: Primers Used for RT-qPCR and CUT&RUN-qPCR.**

| **Purpose** | **Gene** | **Sequence** |
| --- | --- | --- |
| RT-qPCR | NICD3-Forward | 5’ GCTGCTGTTGGACCACTTTG 3’ |
|  | NICD3-Reverse | 5’ CTCTTCTTGGACCCCGACTG 3’ |
|  | SMAD3-Forward | 5’ TGGACGCAGGTTCTCCAAAC 3’ |
|  | SMAD3-Reverse | 5’ CCGGCTCGCAGTAGGTAAC 3’ |
|  | SMAD2-Forward | 5’ CCGACACACCGAGATCCTAAC 3’ |
|  | SMAD2-Reverse | 5’ GAGGTGGCGTTTCTGGAATATAA 3’ |
|  | SMAD4-Forward | 5’ CCACCAAGTAATCGTGCATCG 3’ |
|  | SMAD4-Reverse | 5’ TGGTAGCATTAGACTCAGATGGG 3’ |
|  | TGFBI-Forward | 5’ ATGACCCTCACCTCTATGTACC 3’ |
|  | TGFBI-Reverse | 5’ CACAGTTCACAGTTACAATCCCA 3’ |
|  | ACTB-Forward | 5’ CATGTACGTTGCTATCCAGGC 3’ |
|  | ACTB-Reverse | 5’ CTCCTTAATGTCACGCACGAT 3’ |
| CUT&RUN-qPCR | TGFBI-P1-Forward | 5’ CCGCTCGCAGCTTACTTAAC 3’ |
|  | TGFBI-P1-Reverse | 5’ AGCAGCCGCACGAAGAG 3’ |
|  | TGFBI-P2-Forward | 5’ GGTGTTCTGGGAATCAGCAC 3’ |
|  | TGFBI-P2-Reverse | 5’ TTCCAAATGGGGACCTTGGG 3’ |
|  | TGFBI-P3-Forward | 5’ CATCATTGGCTTGGGCTACG 3’ |
|  | TGFBI-P3-Reverse | 5’ CTGGGGAGACACAGATGGATT 3’ |

| **Name** | **Molecular Weight** | **Docking Score** | **Ligand Efficiency (kcal/mol)** | **Molecular Formula** |
| --- | --- | --- | --- | --- |
| 1,3,6-Tri-O-galloyl-beta-D-glucose | 636.482 | -17.136 | -0.381 | C_27_H_24_O_18_ |
| Depressine | 688.643 | -16.081 | -0.335 | C_30_H_40_O_18_ |
| Forsythoside H | 624.601 | -14.387 | -0.327 | C_29_H_36_O_15_ |
| Pentagalloylglucose | 940.697 | -14.265 | -0.213 | C_41_H_32_O_26_ |
| 1,2,3,6-Tetragalloylglucose | 788.589 | -14.166 | -0.253 | C_34_H_28_O_22_ |
| Eriocitrin | 596.547 | -14.146 | -0.337 | C_27_H_32_O_15_ |
| Echinacoside | 786.745 | -13.662 | -0.248 | C_35_H_46_O_20_ |
| Hesperidin | 610.574 | -13.639 | -0.317 | C_28_H_34_O_15_ |
| Mulberroside A | 568.537 | -13.131 | -0.328 | C_26_H_32_O_14_ |

**Table S3: The list of small-molecule compounds with absolute docking scores >13.**
